# Enhanced remyelination during late pregnancy: involvement of the GABAergic system

**DOI:** 10.1038/s41598-019-44050-4

**Published:** 2019-05-22

**Authors:** Samah Kalakh, Abdeslam Mouihate

**Affiliations:** Department of Physiology, Faculty of Medicine, Health Sciences Centre, Kuwait University, Kuwait City, Safat, 13110 Kuwait

**Keywords:** Multiple sclerosis, Multiple sclerosis

## Abstract

Pregnant women with MS experience fewer relapses, especially during the third trimester. In this study, we explore the cellular and molecular events that bring about the protective effect of late pregnancy on the course of de/remyelination in rats. Using cellular, molecular, and ultrastructural methods, we explored remyelination in response to a focal demyelination in the corpus callosum of late pregnant, virgin, and postpartum rats. We further explored the role of GABA_A_ receptor (GABA_A_R) in the promyelinating effect observed during late pregnancy. Remyelination in response to a gliotoxin-induced demyelination in the corpus callosum was enhanced in late pregnant rats when compared to that seen in virgin and postpartum rats. This pregnancy-associated promyelinating effect was lost when either the GABA_A_R was blocked or when 5α-reductase, the rate limiting enzyme for the endogenous GABA_A_R activator allopregnanolone, was inhibited. Taken together, these data suggest that the pregnancy-associated pro-myelination operates, at least in part, through a GABAergic activated system.

## Introduction

Oligodendrocytes produce an extensive cell membrane called myelin that enwraps axons of neurons within the central nervous system and enhances the speed of conduction of neuronal signals^1^. The loss of oligodendrocytes and the consequent degeneration of this insulating membrane impairs sensory-motor functions of the brain. This demyelination process is typically manifested in patients with multiple sclerosis (MS)^1^.

Clinical evidence reported a dramatic reduction in the number of active lesions in pregnant MS patients^2^. MS patients show an 80% decrease in relapse rate during the third trimester of pregnancy compared to pre-pregnancy^3^. This improvement is higher than what current MS treatments offer (30–60% reduction in relapse rate)^4–6^.The cellular and molecular mechanisms underlying changes in MS course during pregnancy are still not completely understood.

Late gestation is characterized by a heightened GABAergic tone in the brain^7^. This GABAergic tone is mainly mediated by the progesterone metabolite allopregnanolone (ALLO)^7^. In fact, the brain levels of this neuroactive steroid increase substantially during the late phase of pregnancy^8^. GABAergic signaling is involved in the regulation of myelination. Indeed, both OPCs and mature oligodendrocytes express functional GABA_A_ receptors^9^. Moreover, dysregulation of GABAergic signaling has been implicated in MS pathogenesis^10,11^. It is possible that the increased pregnancy-associated GABAergic tone exerts a beneficial effect in demyelinating diseases. It is unclear whether this GABAergic tone could reduce the onset of new demyelinating lesions or promotes remyelination.

Demyelination is associated with a local immune response that takes place within the demyelination area. This local immune response is manifested by activation of microglia and astrocytes^12^. These cells produce a number of pro-inflammatory and anti-inflammatory cytokines^13^. Activated microglia are generally classified into two different phenotypes. The first type is pro-inflammatory microglia which are characterized by the expression inducible nitric oxide synthase (iNOS) and produce pro-inflammatory cytokines such as TNF-α, IL-1β. The second type are immunoregulatory microglia which express the marker arginase-1 (Arg-1) and produce anti-inflammatory cytokines such as IL-4 and IL-10^13,14^.

This gliosis is an essential process required for successful recovery following demyelination^15,16^. Both microglia and astrocytes express GABA_A_R and therefore are potential targets of GABAergic modulation^17,18^. The impact of high GABAergic tone during pregnancy on the activation of these cells following demyelination is still unknown.

In the present study, we used a well-established model of focal demyelination (lysolecithin-induced demyelination in the corpus callosum), and showed that cellular, molecular and ultrastructural manifestations of remyelination were enhanced during late pregnancy, when compared to those of virgin or postpartum rats. More importantly, this pregnancy-associated remyelination was diminished when GABA_A_ receptor (GABA_A_R) was blocked.

## Results

### Remyelination is enhanced following lysolecithin-induced demyelination during pregnancy

**Figure 1:**
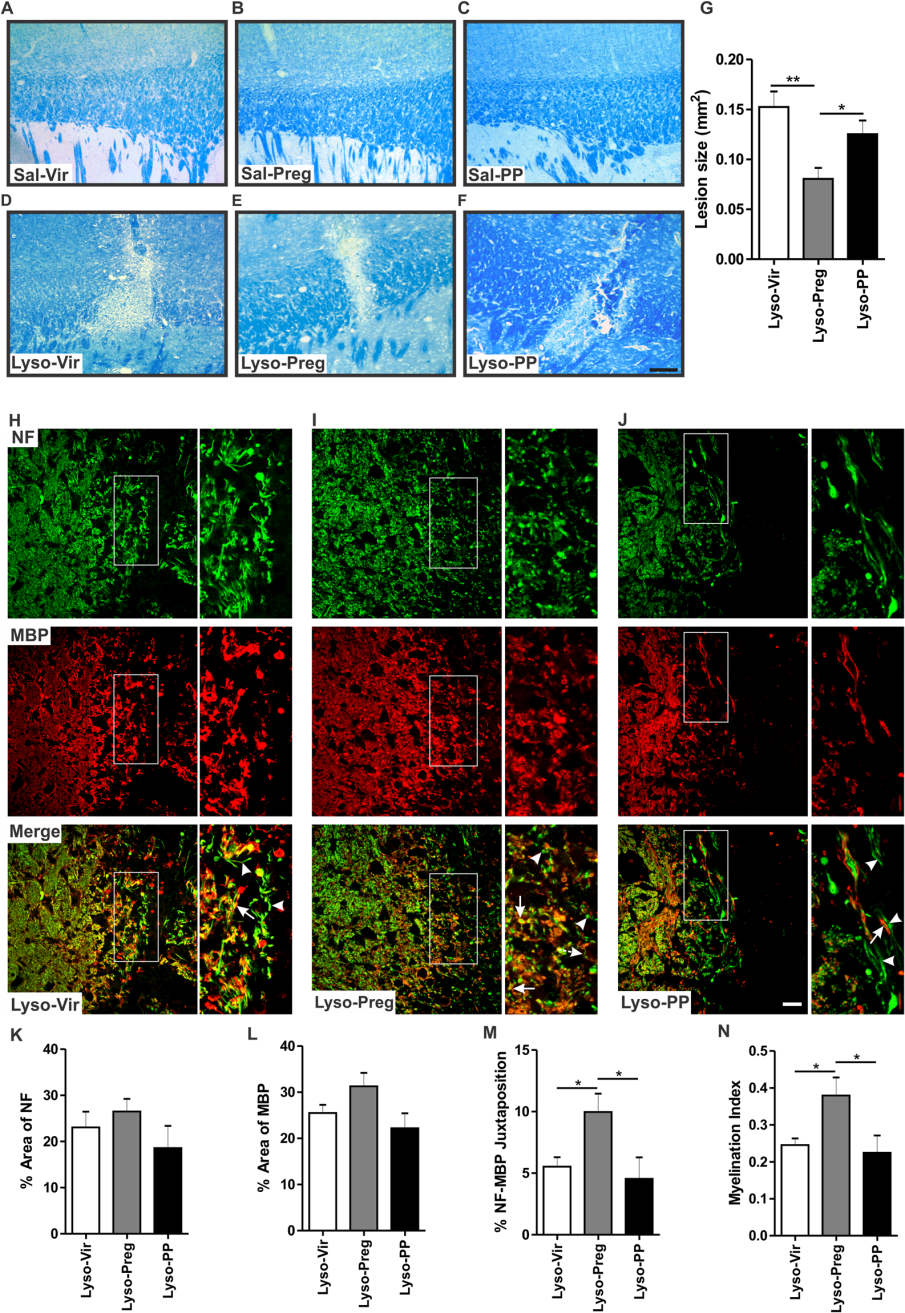
Evaluation of lesion size and axonal integrity 7 days following lysolecithin-induced demyelination. (**A**–**C**) show representative images of LFB staining in saline-injected corpora callosa of virgin, pregnant, and postpartum animals respectively. (**D**–**F**) show representative images of LFB staining in lysolecithin-injected corpora callosa. (**G**) The bar graph shows the demyelination lesion size in the three lysolecithin-injected groups. Pregnant animals had a smaller lesion size compared to both the virgin group (Lyso-Vir: n = 6, Lyso-Preg: n = 6, *p* < 0.01) and the postpartum group (Lyso-PP: n = 5, *p* < 0.05). (**H**–**J**) show immunofluorescent images of NF (green) and MBP (red) at the edge of the demyelination lesion in virgin, pregnant, and postpartum animals respectively. Arrowheads indicate unmyelinated axons while arrows indicate myelinated ones. There was no significant difference in either the percentage area covered by NF^+^ fibers (**K**) or the percentage area covered by MBP^+^ fibers (**L**) between the experimental groups (Lyso-Vir: n = 5, Lyso-Preg: n = 4, Lyso-PP: n = 4, *p* > 0.05). (**M**) Pregnant animals had a significantly higher percentage of juxtaposed NF^+^ and MBP^+^ fibers compared to both virgin and postpartum animals (*p* < 0.05). (**N**) Pregnant animals had a significantly higher myelination index compared to virgin and postpartum animals (*p* < 0.05). Data are represented as mean ± SEM. Scale bar in LFB data = 200 µm. Scale bar in immunofluorescence data = 50 µm.

Saline injection (Sal-Vir n = 4, Sal-Preg n = 4, Sal-PP n = 4) did not result in any damage in the corpus callosum 7 days post-injection (Fig. 1A–C). In contrast, lysolecithin-injected corpora callosa showed evident demyelination (Fig. 1D–F). The size of the lysolecithin-induced demyelination lesion was significantly reduced in pregnant rats when compared to that seen in virgin (*p* < 0.01) and postpartum rats (Lyso-Vir: n = 6, Lyso-Preg: n = 6, Lyso-PP: n = 5, *p* < 0.05, Fig. 1G). There was no significant difference in the size of the demyelination lesion between virgin and postpartum animals.

To assess whether this reduced myelin lesion in pregnant rats is due to reduced demyelination or enhanced remyelination, we evaluated the lesion size at the peak of demyelination 3 days post-demyelination insult^16^. We observed that the size of the demyelination lesion was not significantly different between the three experimental groups 3 days post-lysolecithin injection [Lyso-Vir: n = 9, Lyso-Preg: n = 8, Lyso-PP: n = 8, *p* > 0.05, Supplementary Data 1 (SD 1A-D)]. This observation suggests that the reduction in myelin lesion during late pregnancy is due to an enhanced remyelination rather than a reduced demyelination.

### The myelination index is increased in pregnant rats

The micrographs in Fig. 1H–J show the co-labeling of axons (NF: green) and the myelin sheath (MBP: red). At the edge of the demyelination lesion, myelinated axons were more apparent in pregnant rats. The highly magnified pictures of merged green and red channels showed that demyelinated axons were present in the corpus callosum of Lyso-PP and Lyso-Vir rats (arrowheads). A quantification of juxtaposed NF^+^ axons and MBP^+^ staining showed that the percent of axons surrounded by visually healthy myelin was significantly increased in Lyso-Preg rats when compared to Lyso-Vir and Lyso-PP (Lyso-vir: n = 5, Lyso-preg: n = 4, Lyso-PP: n = 4, *p* < 0.05, Fig. 1M). Similarly, the myelination index, calculated by dividing the value of co-localized NF^+^ and MBP^+^ fraction by the value NF^+^ fraction, was higher in Lyso-Preg rats (*p* < 0.05, Fig. 1N). Investigation of myelination index at 3 days post-demyelination showed no difference between virgin, pregnant, or postpartum rats, further confirming the enhanced remyelination in pregnant rats (Lyso-Vir: n = 8, Lyso-Preg: n = 8, Lyso-PP: n = 7, *p* > 0.05, SD 1 E-K).

### Increased proliferation of OPCs in the demyelinated corpus callosum of pregnant rats

**Figure 2:**
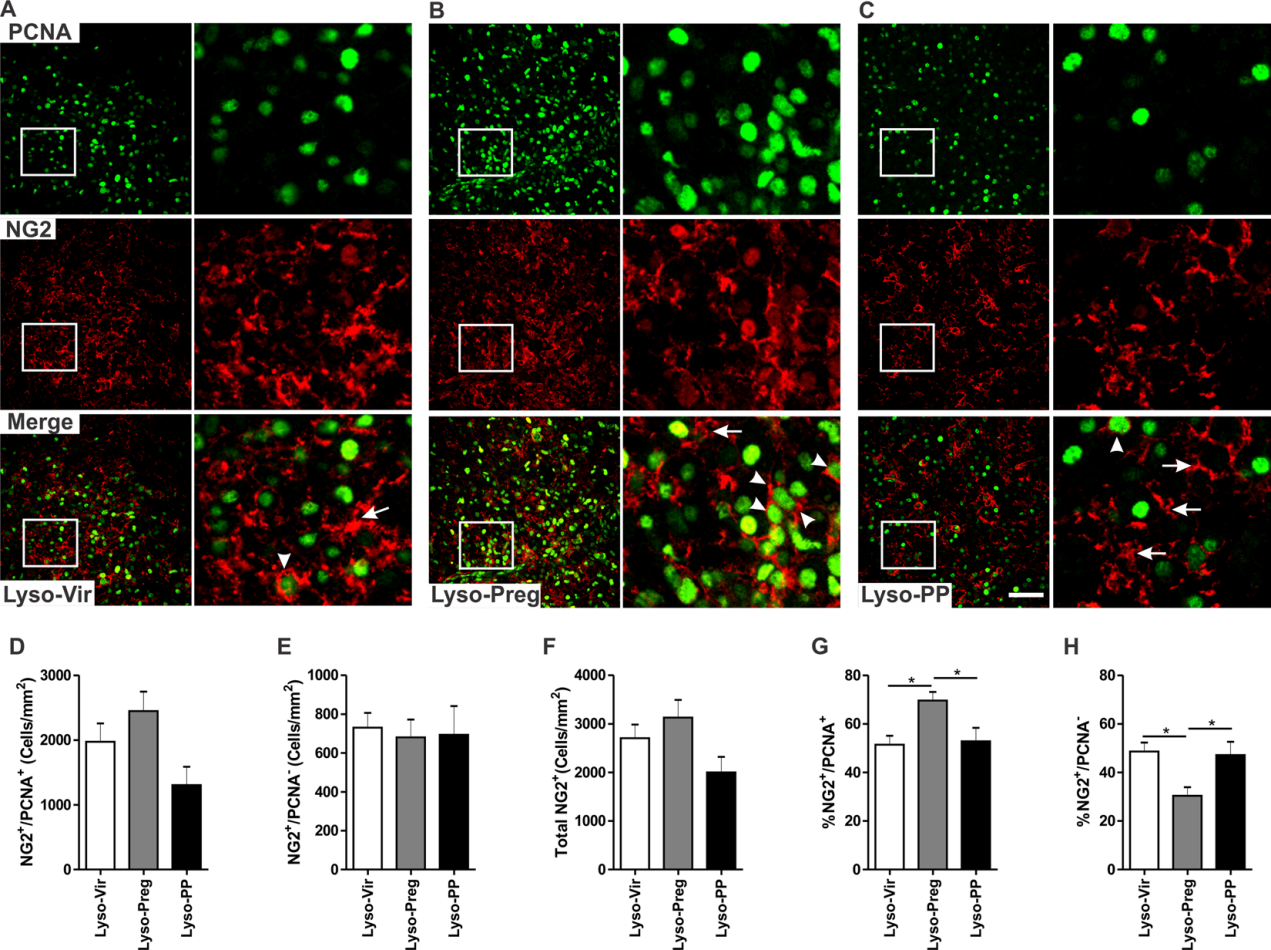
Division of OPCs is enhanced in the vicinity of the corpus callosum 7 days following lysolecithin-induced demyelination in pregnant rats. (**A**–**C**) are immunofluorescent staining images of PCNA (green) and NG2 (red) in lysolecithin-injected corpora callosa of virgin, pregnant, and postpartum animals respectively. (**D**) The bar graph shows that there was no significant difference in the cell density of dividing OPCs (NG2^+^/PCNA^+^, arrowheads), non-dividing OPCs (NG2^+^/PCNA^−^, arrows) (**E**), or the total number of NG2^+^ cells (**F**) between the three experimental groups (Lyso-Vir: n = 6, Lyso-Preg: n = 5, Lyso-PP: n = 4, *p* > 0.05). (**G**) The bar graph shows that focally demyelinated corpus callosa of pregnant animals have a significantly higher percentage of NG2^+^/PCNA^+^ cells when compared to those seen in either virgin or postpartum animals (*p* < 0.05). (**H**) The bar graph shows that demyelinated corpus callosa of pregnant animals had a significantly lower percentage of NG2^+^/PCNA^−^ cells compared to those seen in either virgin or postpartum animals (*p* < 0.05). Data are presented as mean ± SEM. Scale bar = 50 µm.

Because OPCs play an important role in the process of remyelination, we explored whether their cell division was affected during pregnancy using double immunofluorescent staining with the cellular proliferation marker PCNA and the OPCs marker NG2. Dividing OPCs co-expressed NG2 and PCNA (Fig. 2A–C, arrowheads), while non-dividing OPCs lacked the expression of PCNA (Fig. 2A–C, arrows). A large number of NG2^+^ cells were observed at the center of the demyelination lesion in the corpus callosum (Fig. 2A–C). There was no significant difference in the cell density of either NG2^+^/PCNA^+^ cells (Fig. 2D), NG2^+^/PCNA^−^ cells (Fig. 2E), or total NG2^+^ cells (Vir: n = 6, Preg: n = 5, PP: n = 4, *p* > 0.05, Fig. 2F). However, the fraction of dividing OPCs (% NG2^+^/PCNA^+^ among total NG2^+^) was significantly increased, while that of non-dividing OPCs was significantly decreased in the lysolecithin-induced demyelination during pregnancy when compared to those seen in either virgin or postpartum rats (*p* < 0.05, Fig. 2G,H).

### Enhanced axonal myelination in pregnant rats

**Figure 3:**
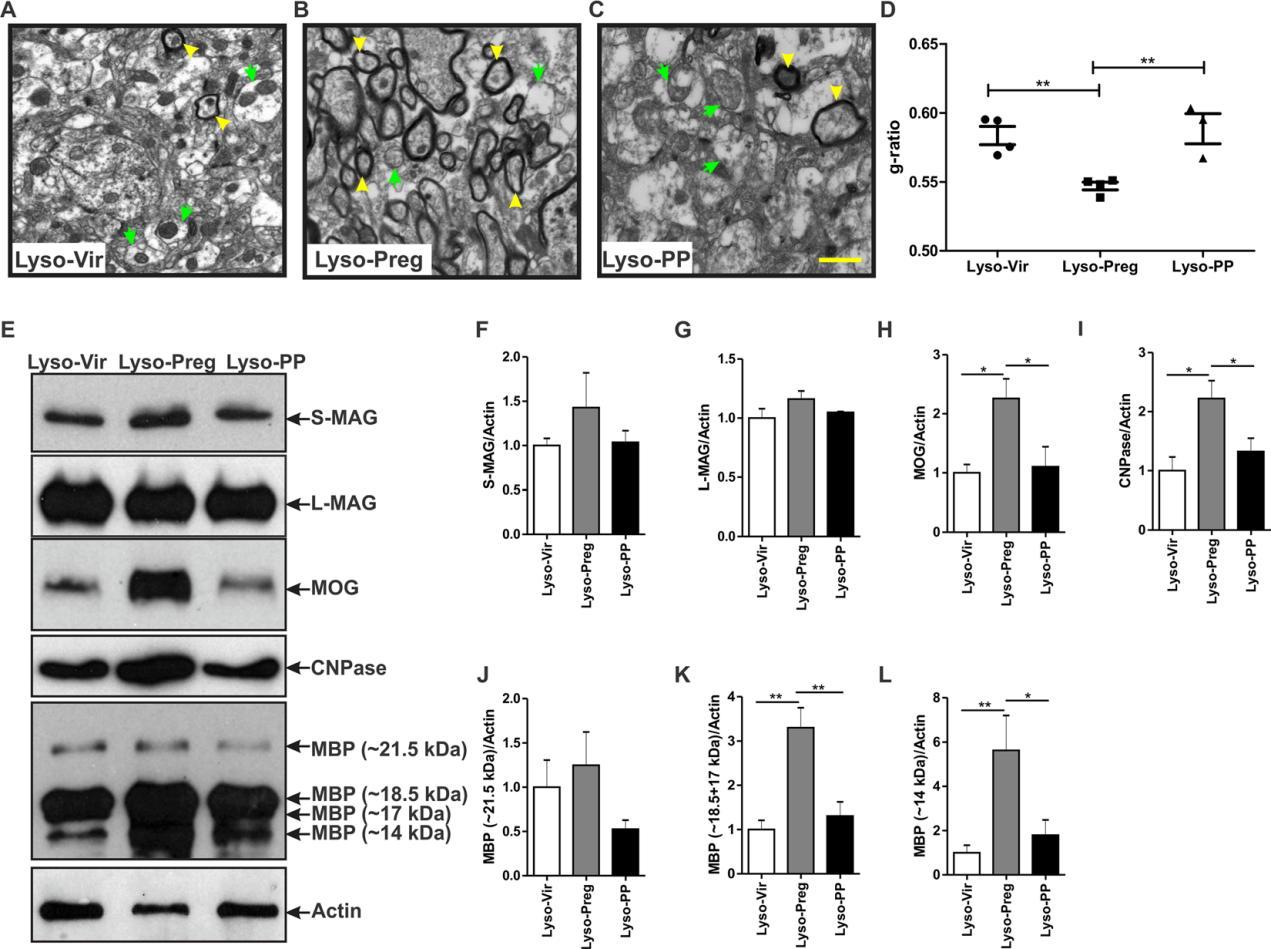
Axonal myelination is increased 7 days post-lysolecithin-induced demyelination in the corpus callosum of pregnant rats. (**A**–**C**) show representative TEM images of the corpus callosum post-lysolecithin injection. Green arrows indicate unmyelinated axons while yellow arrowheads indicate the myelinated ones. (**D**) Graph shows comparison of the g-ratio between the three experimental groups. The axons seen in the corpus callosum of pregnant rats had a significantly lower g-ratio when compared to those seen in virgin and postpartum animals (Lyso-Vir: n = 4, Lyso-Preg: n = 4, Lyso-PP: n = 3, *p* < 0.01). (**E**) Representative figures of western blot for the different myelin proteins. No significant difference was detected in the expression of S-MAG (**F**) or L-MAG (**G**) in demyelinated corpora callosa of the three experimental groups (Lyso-Vir: n = 6, Lyso-Preg: n = 5, Lyso-PP: n = 4, *p* > 0.05). The expression of both MOG (**H**) and CNPase (**I**) were significantly higher in the pregnant rats when compared to those seen in either virgin or postpartum rats (*p* < 0.05). The expression of the 21.5 kDa MBP isoform (**J**) was not different between the three experimental groups (*p* > 0.05). Pregnant rats had a significantly higher expression of 18.5 + 17 kDa isoforms (**K**) when compared to either virgin or postpartum rats (*p* < 0.01). The expression of the 14 kDa MBP isoform (**L**) was significantly increased in pregnant rats when compared to either virgin (*p* < 0.01) or postpartum (*p* < 0.05) rats. Results are presented as mean ± SEM. Scale bar = 1 µm.

The myelin sheath was further assessed at the ultrastructural level using transmission electron microscopy (TEM). Lysolecithin injection into the corpus callosum led to a strong decrease in the myelinated axons in virgin and postpartum rats (Fig. 3A,C) when compared to that seen during pregnancy (Fig. 3B). The g-ratio was significantly reduced in pregnant rats compared to either virgin or postpartum rats (Lyso-Vir: n = 4, Lyso-Preg: n = 4, Lyso-PP: n = 3, Fig. 3D), indicating an enhanced axonal myelination during pregnancy.

### The expression of myelin proteins is higher in focally demyelinated corpus callosum of pregnant rats

The remyelination process involves an increase in the expression of a number of myelin proteins^19^. We explored the expression of 4 major myelin proteins in the vicinity of demyelination area, namely MAG, MOG, CNPase, and MBP^20^. The anti-MAG antibody detected two isoforms; small-MAG (S-MAG) and large-MAG (L-MAG)^21^. There was no significant difference in the expression of these two MAG isoforms (Fig. 3E–G) in the demyelinated corpus callosum in the three physiological states (Lyso-Vir: n = 6, Lyso-Preg: n = 5, Lyso-PP: n = 4, *p* > 0.05). Interestingly, pregnant animals showed a significantly higher levels of the myelin proteins MOG (*p* < 0.05, Fig. 3E,H) and CNPase when compared to virgin and postpartum animals (*p* < 0.05, Fig. 3E,I). We also explored the expression level of the 4 main isoforms of MBP, namely the 21 kDa, 18.5 kDa, 17 kDa, and 14 kDa isoforms. There was no significant difference in the expression of the 21 kDa isoform between the three experimental groups (Fig. 3E,J). On the other hand, a significant increase was observed in the expression of 18.5-17 kDa (*p* < 0.01, Fig. 3E,K), and 14 kDa (*p* < 0.05, Fig. 3E,L) in the demyelinated corpus callosum of pregnant rats compared to those seen in either virgin or postpartum rats. It should be noted that the 18.5-17 kDa isoforms showed a “fused” band in the immunoblot, hence their relative optical densities were combined in the semi-quantification analysis.

### Pregnancy does not alter the local inflammatory response following demyelination

**Figure 4:**
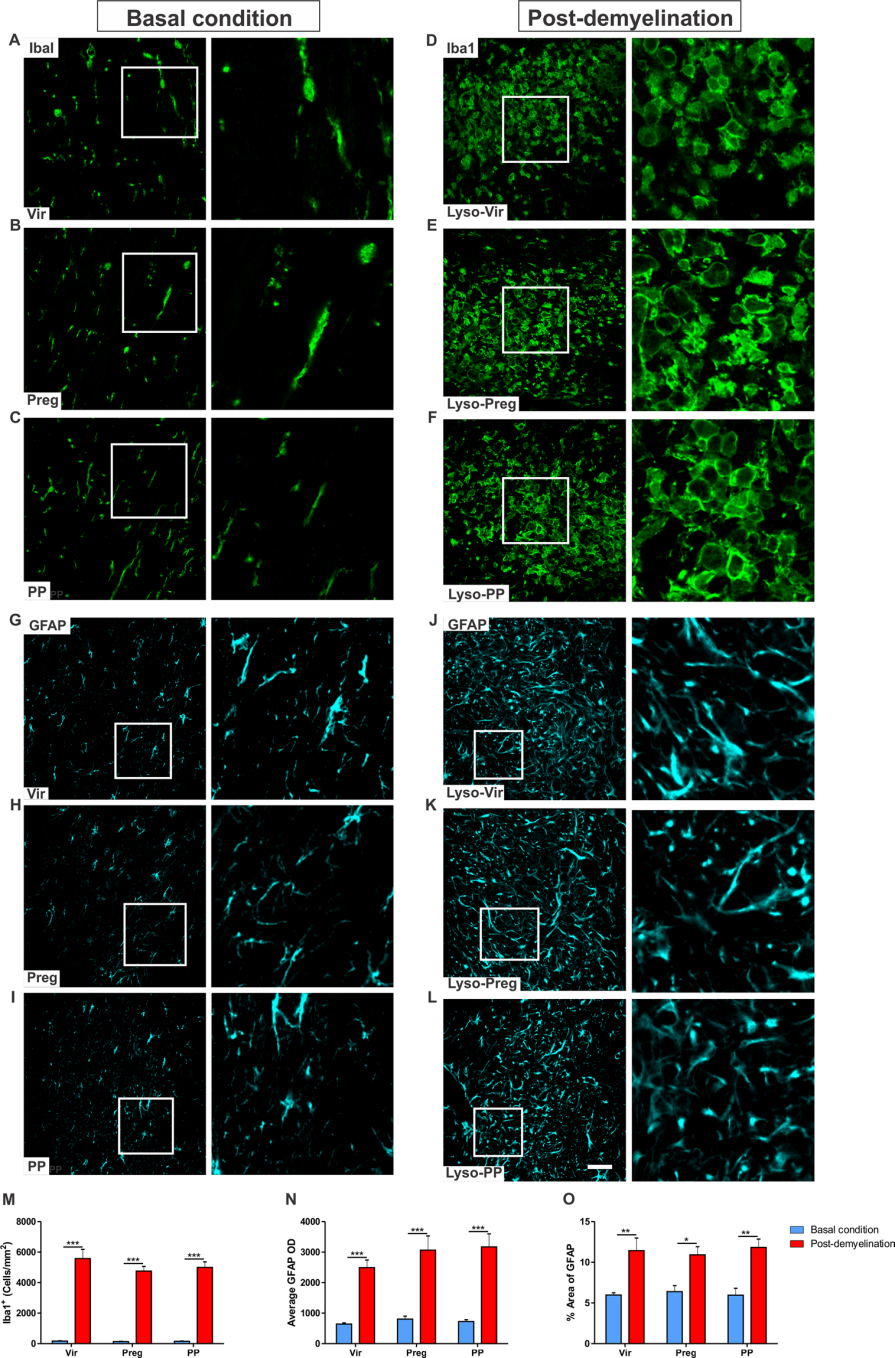
Pregnancy does not affect the local inflammatory response following demyelination in the corpus callosum. (**A**–**C**) are representative immunofluorescent staining images of Iba1 in the corpus callosum in basal condition. (**E**–**G**) demonstrate microglial activation following of lysolecithin injection into the corpora callosa of virgin, pregnant, and postpartum animals respectively 7 days post-demyelination. (**M**) There was no difference in the density of Iba1^+^ cells in the lysolecithin-injected corpus callosum of virgin, pregnant, or postpartum animals (Lyso-Vir: n = 4, Lyso-Preg: n = 6, Lyso-PP: n = 5, *p* > 0.05). (**G**–**I**) are representative immunofluorescent staining images of GFAP in the corpus callosum in basal condition. (**J**–**L**) shows activated GFAP^+^ cells following of lysolecithin injection into the corpora callosa of virgin, pregnant, and postpartum animals respectively 7 days post-demyelination. (**N**) There was no significant difference in the OD of GFAP^+^ staining between virgin, pregnant, or postpartum animals (Lyso-Vir: n = 4, Lyso-Preg: n = 6, Lyso-PP: n = 5, *p* > 0.05). (**O**) The fraction area covered by GFAP^+^ cells was not different between the three experimental groups following demyelination (*p* > 0.05).

Microglial activation was prominent at the centre of the demyelination lesion of all experimental groups (Lyso-Vir: n = 4, Lyso-Preg: n = 6, Lyso-PP: n = 5, Fig. 4D–F) when compared to that seen in the uninjured corpus callosum (Vir: n = 5, Preg: n = 4, PP: n = 5, Fig. 4A–C). Indeed, the demyelination induced a significant increase in microglial cell density (Fig. 4M; *p* < 0.001). However, this increase in microglial cell density was not significantly different between virgin, pregnant, or postpartum animals (Fig. 4M; *p* > 0.05).

Activation of astrocytes was also increased in the lysolecithin-induced demyelination in the corpus callosum (Lyso-Vir: n = 4, Lyso-Preg: n = 6, Lyso-PP: n = 5, Fig. 4J–L) when compared to that seen in basal condition (Vir: n = 4, Preg: n = 5, PP: n = 4, Fig. 4G–I). There was no difference in the OD of GFAP^+^ cells (*p* > 0.05, Fig. 4N) or the density of GFAP^+^ cells (*p* > 0.05, Fig. 4O) between the three experimental groups.

To further assess the activation state of microglia, we investigated the expression of the pro-inflammatory microglial marker iNOS, and the regulatory microglial marker arginase-1 in the demyelinated area of the corpus callosum. The expression of these markers was not different between the three experimental groups neither at the peak of inflammation 3 days post-demyelination, nor at the start of remyelination 7 days post-demyelination (SD 2A-D).

In addition to their morphological changes when activated by a demyelinating insult, microglia, and to lesser extent astrocytes, produce a set of pro- and anti-inflammatory cytokines. Therefore, we measured the concentration of pro- and anti-inflammatory cytokines (IL-1β, TNF-α, IL-4, IL-10) at the centre of the demyelination lesion at 3 days and 7 days post demyelination insult. The concentration levels of IL-1β were significantly higher at 3 days (3D) post-demyelination insult when compared to 7 days (7D) post-demyelination insult in the corpora callosa of virgin (*p* < 0.01), pregnant (*p* < 0.001), and postpartum (*p* < 0.001) animals [3D (Lyso-Vir: n = 7, Lyso-Preg: n = 8, Lyso-PP: n = 8), 7D (Lyso-Vir: n = 5, Lyso-Preg: n = 5, Lyso-PP: n = 4)] (SD 2E). The concentration levels of TNF-α were not different between the three experimental groups at either 3 or 7 days post-demyelination insult (*p* > 0.05) (SD 2F). The concentration levels of IL-4 were significantly higher at 7 days post-demyelination insult when compared to those seen at 3 days post-demyelination insult in the virgin (*p* < 0.05), pregnant (*p* < 0.05), and postpartum (*p* < 0.001) rat groups (SD 2G). Levels of IL-10 on the other hand did not change over time in the three experimental groups (*p* > 0.05) (SD 2H). Furthermore, there was no significant effect of the physiological state (virgin, pregnant, postpartum) on the expression level of IL-1β (*p* > 0.05), TNF-α (*p* > 0.05), or IL-10 (*p* > 0.05) (SD 2E, 2F, 2H). However, we have noticed that the concentration levels of IL-4 were significantly reduced in the corpus callosum of postpartum animals at 3 days post demyelination lesion when compared to their corresponding values in either virgin or pregnant animals (*p* < 0.05) 3 days post-demyelination (SD 2G).

### GABA_A_R blockade worsens the demyelination lesion in pregnant rats

**Figure 5:**
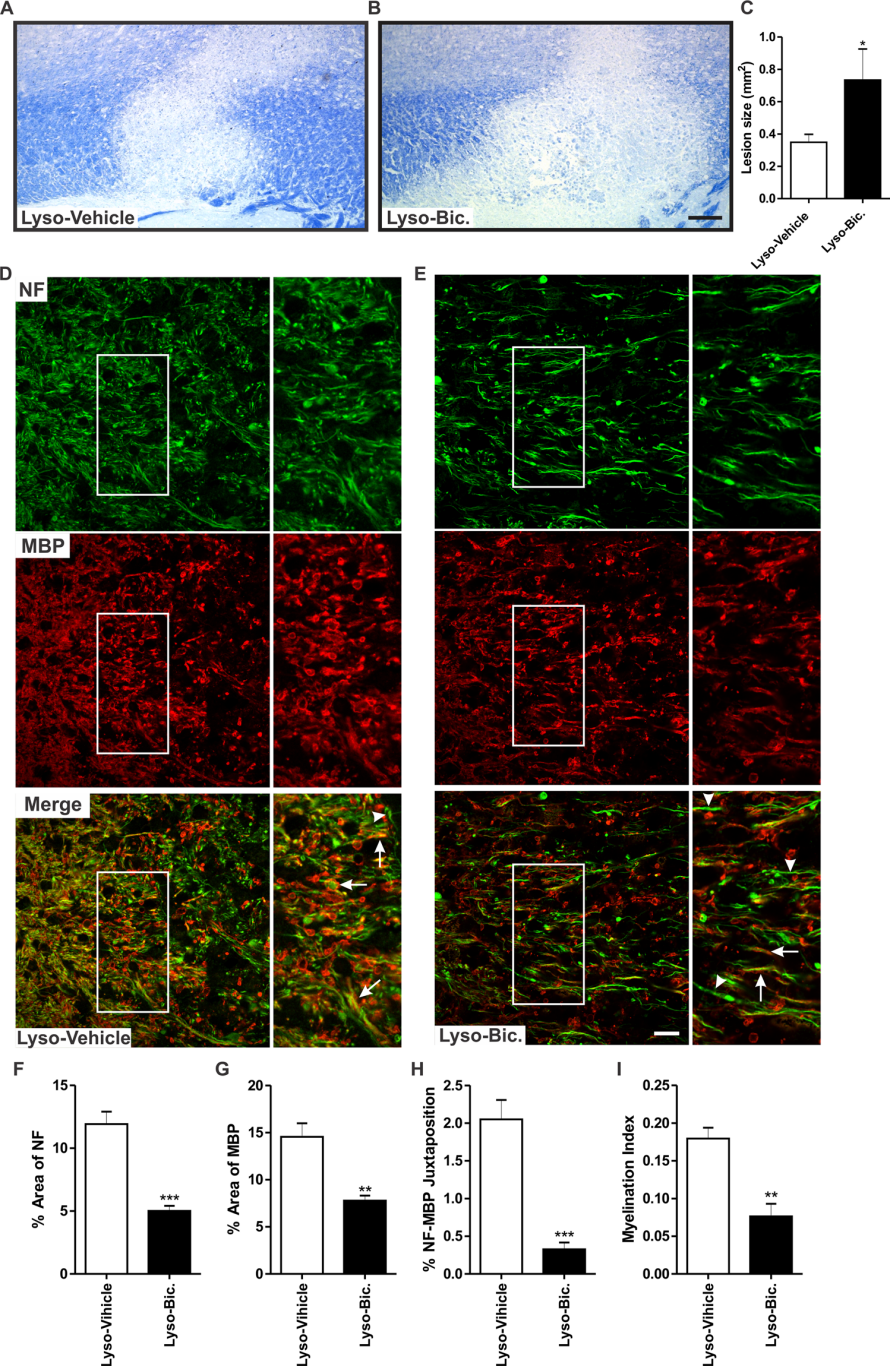
Pharmacological blockade of GABA_A_R exacerbates demyelination lesion and reduces axonal integrity following 7 days lysolecithin-induced demyelination in the corpus callosum of pregnant rats. (**A**,**B**) show representative images of LFB staining in lysolecithin-injected corpora callosa of vehicle-treated and Bic-treated pregnant rats. Graph bar in (**C**) shows that administration of Bic resulted in a significantly larger demyelination lesion when compared to vehicle-treated rats (Lyso-Vehicle: n = 7, Lyso-Bic: n = 5, *p* < 0.05). (**D**,**E**) are immunofluorescent staining images of NF (green) and MBP (red) in the corpora callosa of vehicle-treated and Bic-treated animals respectively. Arrowheads indicate unmyelinated axons while arrows indicate myelinated ones. Bic treatment resulted in a significant reduction in the percentage area covered by NF^+^ fibers (*p* < 0.0002) (**F**), in the percentage area covered by MBP^+^ fibers (*p* < 0.002) (**G**), in the percentage area of juxtaposed NF^+^ and MBP^+^ fibers (*p* < 0.0002) (**H**), and in the myelination index (*p* < 0.002) (**I**) when compared to vehicle-treated animals (Lyso-Vehicle: n = 5, Lyso-Bic: n = 5). Results are presented as mean ± SEM. Scale bar in LFB micrograph = 200 µm. Scale bar in immunofluorescence micrograph = 50 µm.

Substantial experimental evidence strongly suggests that GABA_A_R activation is involved in the process of remyelination^22,23^. We reasoned that if the increased GABAergic tone during late pregnancy underlies the observed enhanced remyelination, this protective effect could be prevented by exogenously administered GABA_A_R antagonist. Therefore, we gave daily injections of either bicuculline (Bic), a GABA_A_R antagonist, or the vehicle (saline) to pregnant rats subjected to focal demyelination. Administration of Bic resulted in a significant increase in the size of the demyelination lesion size when compared to vehicle-treated animals (Lyso-Vehicle: n = 7, Lyso-Bic: n = 5, *p* < 0.05, Fig. 5A–C).

We also assessed the effect of Bic treatment on axonal integrity at the edges of the demyelination lesion (Lyso-Vehicle: n = 5, Lyso-Bic: n = 5, Fig. 5D,E). Bic significantly reduced the density of NF^+^ fibers (*p* < 0.0002, Fig. 5F), the density of MBP^+^ fibers (*p* < 0.002, Fig. 5G), the percentage area where NF^+^ and MBP^+^ are juxtaposed (*p* < 0.0002) (Fig. 5H), and the myelination index (*p* < 0.002, Fig. 5I).

### GABA_A_R blockade reduces the density of OPCs in the demyelinated area of pregnant rats

**Figure 6:**
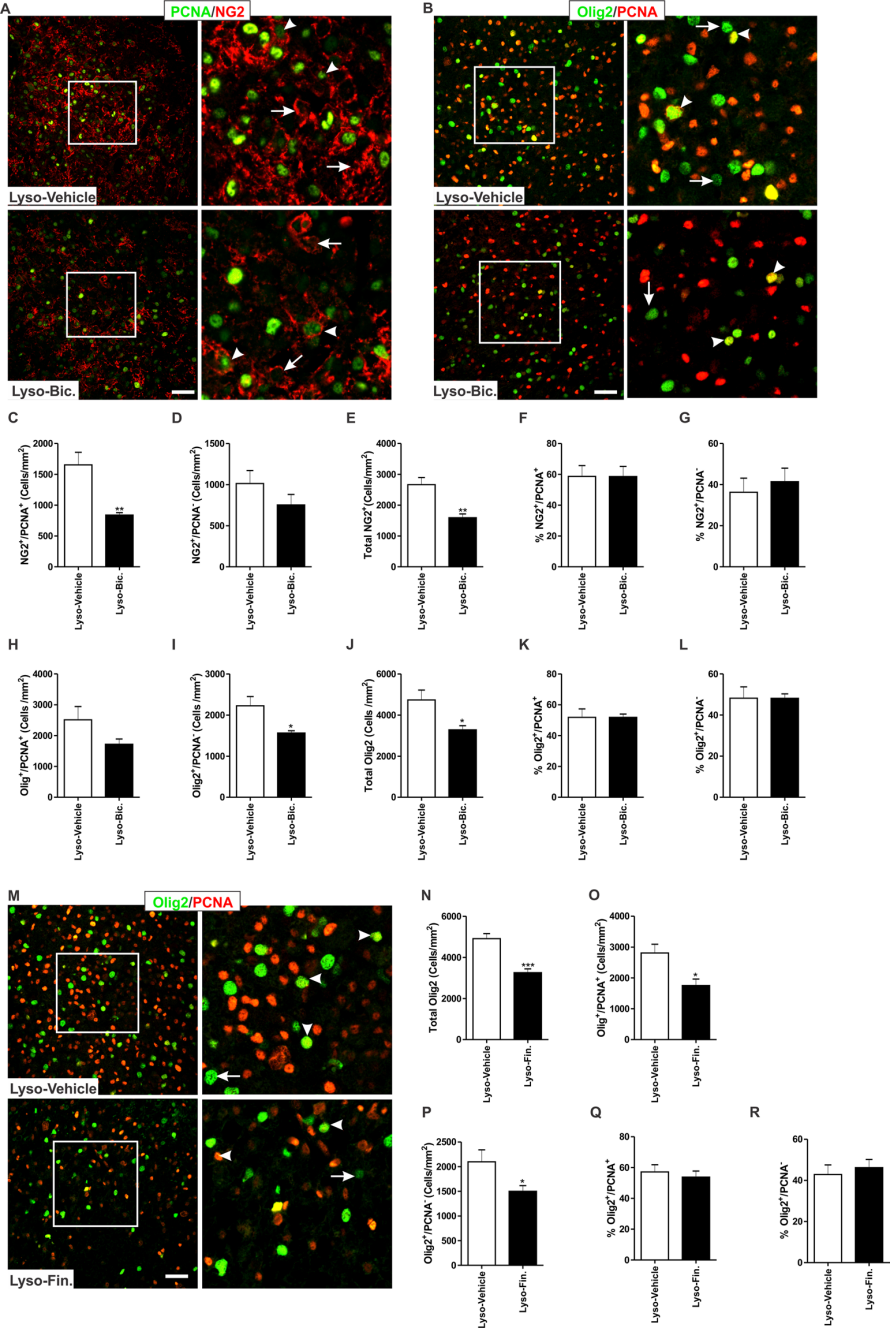
Impact of Bic on the number of OPCs 7 days following lysolecithin-induced demyelination in the corpus callosum of pregnant rats. (**A**) shows representative images of the proliferation marker PCNA (green) and the OPC marker NG2 (red), while (**B**) shows representative images of PCNA (green) and pan oligodendrocyte marker Olig2 (OPCs and mature oligodendrocytes; red) in the demyelinated corpus callosum of vehicle-treated and Bic-treated rats respectively. (**C**) Bic treatment resulted in a significant reduction in the number of NG2^+^/PCNA^+^ cells (arrowheads) in the vicinity of the demyelinated corpus callosum when compared to vehicle-treated animals (Lyso-Vehicle: n = 4, Lyso-Bic: n = 4, *p* < 0.01). (**D**) There was no significant difference in the cell density of NG2^+^/PCNA^−^ cells (arrows) between vehicle-treated and Bic-treated animals (*p* > 0.05). (**E**) The total number of NG2^+^ cells was significantly reduced by Bic treatment in comparison to vehicle treatment (*p* < 0.01). There was no significant difference in the fraction of NG2^+^/PCNA^+^ (**F**), or the fraction of NG2^+^/PCNA^−^ (**G**) cells (*p* > 0.05). (**H**) There was no significant difference in the density of Olig2^+^/PCNA^+^ cells (Olig2/red, PCNA/green) between vehicle-treated and Bic-treated animals (Lyso-Vehicle: n = 4, Lyso-Bic: n = 5, *p* > 0.05). Bic administration resulted in a significant reduction in the density of Olig2^+^/PCNA^−^ cells (**I**) and total Olig2^+^ cells (**J**) in the vicinity of the demyelinated corpus callosum when compared to vehicle-treated animals (*p* < 0.05). There was no significant difference in the percentage of Olig2^+^/PCNA^+^ cells (**K**) or Olig2^+^/PCNA^−^ cells (**L**) between vehicle-treated and Bic-treated animals (*p* > 0.05). Results are presented as mean ± SEM. Scale bar = 50 µm.

Administration of Bic to pregnant rats subjected to focal demyelination led to a significant reduction in the cell density of NG2^+^/PCNA^+^ cells (Lyso-Vehicle: n = 4, Lyso-Bic: n = 4, *p* < 0.01, Fig. 6A,C), but not that of NG2^+^/PCNA^−^ cells (*p* > 0.05, Fig. 6D) within the area of the demyelination lesion. Bic injection resulted in a significant reduction in the cell density of total NG2^+^ cells in the vicinity of the demyelination lesion compared to vehicle-treated animals (*p* < 0.01, Fig. 6E). Administration of Bic did not affect the fraction of either dividing (*p* > 0.05, Fig. 6F) or non-dividing NG2^+^ cells (*p* > 0.05, Fig. 6G). We further explored the effect of Bic using a pan oligodendrocyte marker which labels both OPCs and mature oligodendrocytes (Olig2). Bic induced a slight, albeit not statistically significant, decrease in the density of Olig2^+^/PCNA^+^ cells (*p* > 0.05, Fig. 6H). Bic administration significantly reduced the density of Olig2^+^/PCNA^−^(*p* < 0.05, Fig. 6I) and total Olig2^+^ cells (*p* < 0.05, Fig. 6J). Bic administration did not impact the fraction of either dividing (*p* > 0.05, Fig. 6K) or non-dividing Olig2^+^ cells (*p* > 0.05, Fig. 6L).

Since the increased GABAergic tone during late pregnancy is mainly mediated by the progesterone metabolite ALLO^7^, we conducted an experiment to assess the contribution of endogenous ALLO on de/remyelination during pregnancy. Under demyelination condition, we inhibited the synthesis of ALLO using the 5α-reductase inhibitor finasteride (Fin). The impact of ALLO synthesis inhibition was similar to that of pharmacological GABA_A_R blockade. Indeed, Fin administration significantly reduced the density of Olig2^+^ cells in the vicinity of the demyelination lesion (Lyso-Vehicle: n = 4, Lyso-Fin: n = 5, *p* < 0.001, Fig. 6M,N). We also explored the density of Olig2^+^ cells expressing the proliferation marker PCNA. Fin administration significantly reduced the density of Olig2^+^/PCNA^+^ cells (*p* < 0.05, Fig. 6O) and Olig2^+^/PCNA^−^ cells (*p* < 0.05, Fig. 6P). However, the percentages of Olig2^+^/PCNA^+^ (Fig. 6Q) or Olig2^+^/PCNA^−^ (Fig. 6R) were not different between the two experimental groups (*p* > 0.05). In addition to its effect on the cell density of oligodendrocytes, administration of Fin resulted in a larger demyelination lesion in the corpus callosum of pregnant rats when compared to demyelination lesion seen in vehicle-treated pregnant rats (Lyso-Vehicle: n = 5, Lyso-Fin: n = 7, *p* < 0.05, SD 3).

### GABA_A_R blockade reduces the expression of myelin-associated glycoprotein in pregnant rats

**Figure 7:**
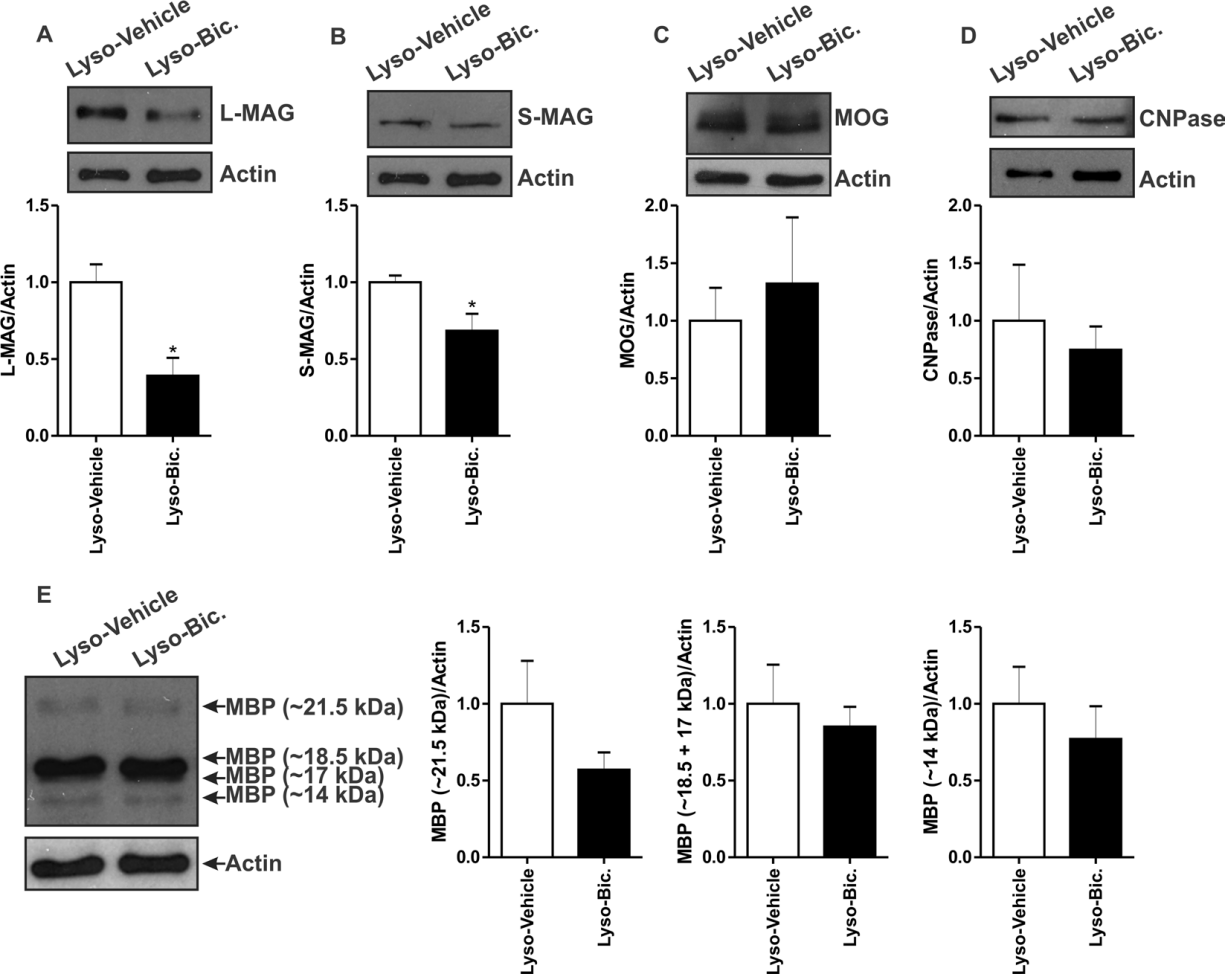
Effect of Bic on myelin proteins expression 7 days following lysolecithin-induced demyelination in the corpus callosum using western blot. Administration of Bic significantly reduced the expression levels of L-MAG (**A**) and S-MAG (**B**) in lysolecithin-injected corpora callosa when compared to those seen in vehicle treated rats (Lyso-Vehicle: n = 4, Lyso-Bic: n = 5, *p* < 0.05). Bic administration on the other hand did not affect the expression of MOG (**C**), CNPase (**D**), or MBP (**E**) when compared to vehicle-treated rats (*p* > 0.05). Results are presented as mean ± SEM.

We further evaluated the expression of myelin associated proteins following the demyelination injury. Administration of Bic resulted in a significant reduction in the expression of both S-MAG and L-MAG in the demyelinated corpus callosum of pregnant rats when compared to vehicle-treated animals (Lyso-Vehicle: n = 4, Lyso-Bic: n = 5, *p* < 0.05, Fig. 7A,B). On the other hand, Bic treatment did not affect the expression of either MOG, CNPase, or MBP when compared to vehicle-treated pregnant rats (*p* < 0.05, Fig. 7C–E).

### GABA_A_R blockade exacerbates microglial activation following demyelination

**Figure 8:**
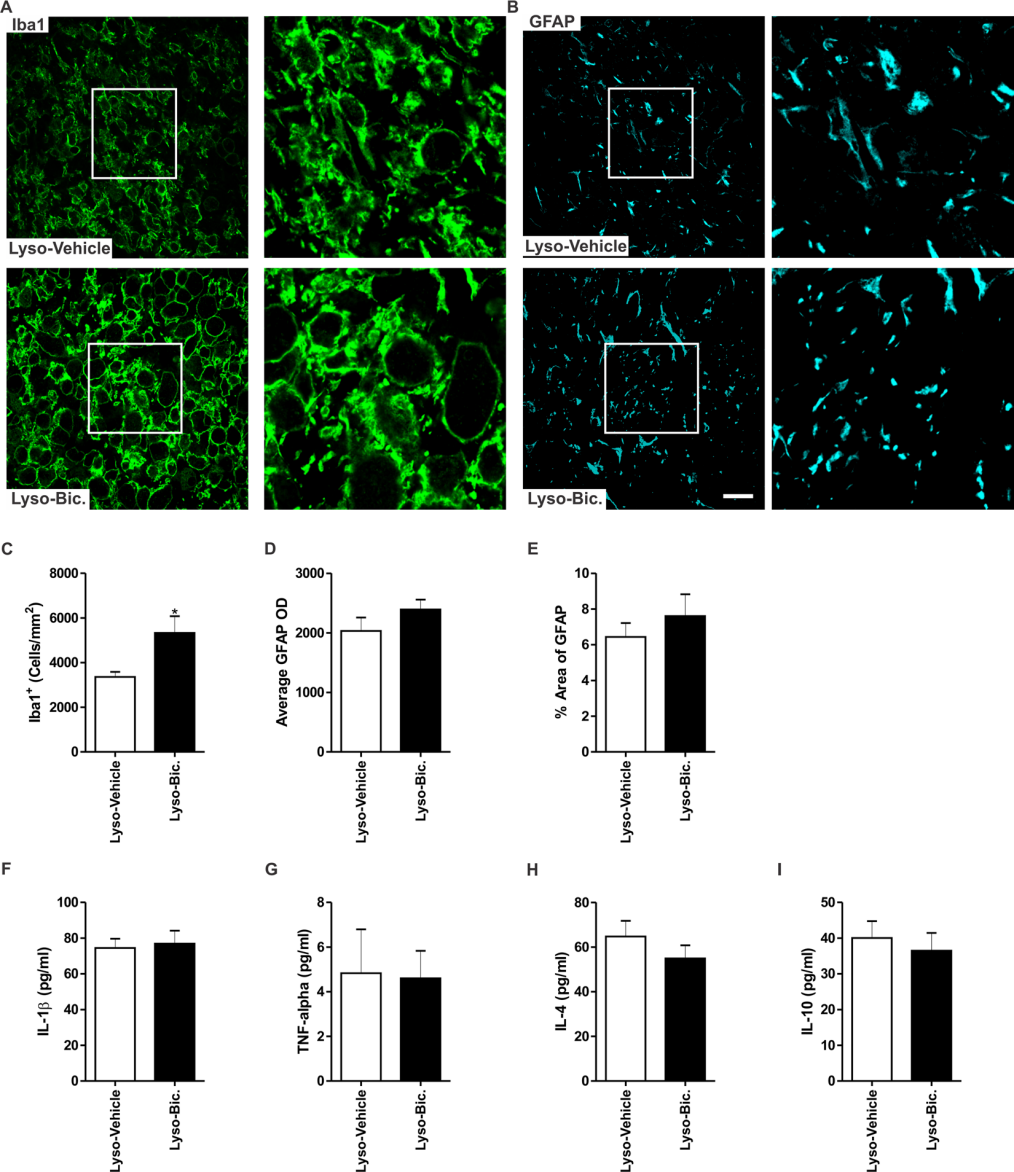
GABA_A_R blockade exacerbates microglial activation 7 days following lysolecithin-induced demyelination in the corpus callosum of pregnant rats. (**A**) shows representative images of Iba1 (green) in the corpus callosum of vehicle-treated and Bic-treated pregnant rats respectively 7 days post-demyelination. (**C**) The density of Iba1^+^ cells was significantly increased following Bic administration (*p* < 0.05). (**B**) shows representative images of GFAP (cyan) in the corpus callosum of vehicle-treated and Bic-treated pregnant rats respectively 7 days post-demyelination. Bic treatment did not affect GDAP OD (**D**) or the percentage area covered by GFAP (**E**) (Lyso-Vehicle: n = 4, Lyso-Bic: n = 4, *p* > 0.05). (**F**–**I**) are bar graphs showing the cytokine measurements in the demyelinated corpus callosum tissue of vehicle-treated and Bic-treated pregnant rats 7 days post-demyelination obtained by multiplex ELISA. There was no significant difference in the expression of any of these cytokines between vehicle-treated and Bic-treated pregnant rats (*p* > 0.05). Results are presented as mean ± SEM. Scale bar = 50 µm.

We investigated the effect of Bic administration on the inflammatory response associated with the demyelination injury in the vicinity of the lesion (Fig. 8A,B). Administration of Bic significantly increased the density of Iba1^+^ cells when compared to vehicle-treated animals (*p* < 0.05, Fig. 8C). On the other hand, Bic did not affect astrocytic activation level, measured by the GFAP OD and the fraction area covered by GFAP^+^ staining, when compared to vehicle-treated rats (Lyso-Vehicle: n = 4, Lyso-Bic: n = 4, *p* > 0.05, Fig. 8D,E).

One of the major functions of microglia is the production of inflammatory cytokines within the demyelinated area of the corpus callosum. Thus, we measured the levels of key pro- (IL-1β, TNF-α) and anti-inflammatory (IL-4, and IL-10) cytokines in the demyelinated corpus callosum of both vehicle-treated and Bic-treated pregnant rats 7 days post-demyelination using multiplex ELISA (Lyso-Vehicle: n = 7, Lyso-Bic: n = 7). Despite its effect on microglial cell density, Bic did not significantly affect the expression levels of any of these cytokines in the demyelinated corpus callosum of pregnant rats (*p* > 0.05, Fig. 8F–I).

### The expression of GABA_A_Rγ2 is upregulated following demyelination in the corpus callosum of pregnant rats

Brain levels of the neuroactive steroid ALLO are markedly increased during late gestation^6^. GABA_A_R is the main mediator of ALLO’s action^8^. The affinity of ALLO to GABA_A_R depends on the receptor’s subunit composition. GABA_A_R containing the γ2 (GABA_A_Rγ2) subunit shows more affinity to ALLO^24^. Therefore, we sought to determine the expression level of this particular subunit in the demyelinated corpus callosum of virgin, pregnant, and postpartum animals using western blot. Two major isoforms of GABA_A_Rγ2 were detected at an apparent molecular weights of ~47 kDa and ~45 kDa (SD 4A). The expression of the ~47 kDa isoform was not significantly different between the three experimental groups (Lyso-Vir: n = 4, Lyso-Preg: n = 4, Lyso-PP: n = 4, *p* > 0.05, SD 4B). On the other hand, pregnant animals had a significantly higher expression level of the ~45 kDa isoform when compared to either virgin or postpartum animals (*p* < 0.05, SD 4C). Using double immunofluorescent staining, we found that subsets of both NG2^+^ cells (OPCs) (SD 4D–F) and Iba1^+^ cells (microglia) (SD 4G–I) express GABA_A_Rγ2 in the vicinity of the demyelination lesion.

## Discussion

In the present study, we show that pregnancy creates a conducive environment for pro-myelination in response to a focal demyelination injury when compared with virgin and postpartum rats. The current experimental evidence suggests that the mechanism underlying such increased pro-myelination during pregnancy is, at least in part, due to enhanced GABAergic tone. Indeed, the blockade of GABA_A_R exacerbated the focal demyelination seen as an increased lesion size in pregnant rats.

Demyelination-associated symptoms are reduced during pregnancy in MS patients and in EAE mice model of demyelination. These remissions appear to be more apparent during the late phase of pregnancy^3,25–27^ and resurge during the postpartum period^28^. The mechanism underlying this remission is unclear. One potential mechanisms by which pregnancy could contribute to the protection against demyelination is through the alteration in the innate immune response and the well-known immune-tolerant state associated with pregnancy^3^. However, experimental evidence suggests that induction of EAE in pregnant rodents did not affect the systemic levels of pro-inflammatory cytokines^27^. Similarly, we did not detect any change in the density of microglia or astrocytes in the vicinity of the demyelination lesion between pregnant and non-pregnant rats (Fig. 4). It appears that the protective effect of pregnancy is not related to the change in its associated immune-tolerant status^3^.

Late pregnancy is characterized by an increase in GABAergic tone^7^. This increase is mainly driven by the increasing levels of the progesterone metabolite allopregnanolone (ALLO) during this period^7^. ALLO extends the opening time of chloride channels of GABA_A_R, therefore enhancing inhibitory neurotransmission^29^. The increased GABAergic tone via ALLO has been shown to suppress the stress response during late pregnancy by attenuating the hypothalamic-pituitary-adrenal axis activation^30^. However, its effect on myelin repair is still unknown. Our study is the first to demonstrate the involvement of increased GABAergic tone in the enhanced remyelination observed during late pregnancy. This is supported by the finding that this effect was lost when GABA_A_R was blocked.

In addition to virgin animals, we compared the remyelination capacity of pregnant animals to that of postpartum animals. The postpartum period is characterized by a reduction in GABAergic signaling^7^. This reduction is mainly due to the sharp reduction in ALLO levels postpartum in both the plasma and the brain^7,8^. Previous evidence showed a decline in the remyelination capacity in postpartum non-breastfeeding MS patients^31^. It is noteworthy that MS patients are advised not to breastfeed during the postpartum as a contraindication of drugs prescribed to manage MS^31^. In the present study, we found that the remyelination in non-lactating postpartum rats was equally reduced. Perhaps this postpartum-associated reduction in remyelination is related to reduced GABAergic tone during postpartum^8,32^.

At the molecular level, pregnant animals showed higher expression levels of CNPase, one of the earliest markers of the remyelination process^33^; MBP, which plays a crucial role in the compaction of different layers of myelin^19^, and MOG; which is a marker of the very late stage of remyelination^20^. The observation that these markers of remyelination also increased in parallel with increased GABAergic tone provides a potential underlying mechanism of the observed amelioration in MS disease severity during late pregnancy.

Interestingly, GABA_A_R blockade in pregnant rats specifically downregulated the expression of MAG. The mechanism underlying this specific effect of GABA_A_R blockade on MAG is unclear. MAG is a myelin protein with a periaxonal localization^34^. It maintains the space between the innermost myelin surface and the axonal surface by binding to axonal gangliosides^35^. Due to this cellular localization, it is possible that the detrimental effect of bicuculline on axons makes MAG the most sensitive protein to the effect of GABA_A_R blockade.

Another essential step for successful remyelination is the recruitment of a sufficient number of OPCs to the site of demyelination^36^. Pregnancy has been previously shown to enhance cellular proliferation during or following demyelination^25^. In fact, induction of demyelination in the corpus callosum using lysolecithin at both early (GD7) and late (GD14) pregnancy resulted in a significant increase in cellular proliferation of pregnant mice compared to virgins. A fraction of these dividing cells expressed the OPC marker NG2^25^.Using double immunofluorescent staining, we observed that the fraction of proliferating OPCs (PCNA^+^) was higher in demyelinated area of pregnant animals when compared to those of virgin and postpartum animals. The enhanced OPCs proliferation observed in pregnant animals is likely due to the altered hormonal milieu during pregnancy namely that of the progesterone metabolite ALLO. Indeed, ALLO has been shown to enhance OPCs proliferation *in vitro* in basal conditions^37,38^. Interestingly, in the present study, the mitotic activity of OPCs in the vicinity of the demyelination lesion was reduced when ALLO production was inhibited or when ALLO receptor was antagonized (Fig. 6), implicating the GABA_A_R in this effect. This is the first *in vivo* study to show a potential role of ALLO in enhancing OPCs proliferation following a demyelination insult during pregnancy. The mechanism through which ALLO enhances OPCs proliferation is still unclear. However, there are indications that ALLO-induced activation of GABA_A_R leads to mobilization of cyclic AMP-responsive element-binding protein 1 (CREB1) within OPCs, which is known to cause the upregulation of genes responsible for cell division^39,40^.

Microglial activation can have beneficial, as well as detrimental effects on remyelination. Beneficial effects of microglia include the clearance of cell debris and promotion of OPCs differentiation via the production of anti-inflammatory cytokines^16^. Activated microglia can also exacerbate tissue damage through different mechanisms including the production of pro-inflammatory cytokines, free radicals, proteases, and excessive phagocytosis^41^. Similarly, astrocytosis can either help or impede the remyelination process. While astrocytosis delimits the lesion and prevents its spreading, it might also impede the migration of OPCs into the demyelination lesion and therefore prevent successful remyelination^13^. We observed that both microglia and astrocytes were highly activated in and around the demyelination lesion. There was no change in the density or the level of activation of microglia or astrocytes in the vicinity of the demyelination lesion between pregnant, virgin, and postpartum rats.

It is well-established that acute inflammation is characterized by higher levels of pro-inflammatory cytokines compared to anti-inflammatory cytokines. This is later followed by a switch into an anti-inflammatory milieu^16^. In our hands, the pro-inflammatory cytokine IL-1β was higher during acute inflammation, while the anti-inflammatory cytokine IL-4 was higher during the start of remyelination, demonstrating a time-dependent shift from pro- to anti-inflammatory response to demyelination insult that takes place in pregnant and non-pregnant rats. While pregnancy is characterized by a shift from a systemic Th1-dominant state into a Th2-dominant state, there was no specific effect of pregnancy on the inflammatory milieu within the lesioned brain, neither at the acute nor at the start of remyelination phase. Indeed, we did not detect any significant change in the expression levels of either pro-inflammatory (IL-1β, TNF-α), or anti-inflammatory cytokines (IL-4, IL-10) in pregnant rats when compared to non-pregnant rats. This discrepancy could be because the immune shift from Th1 to Th2 during pregnancy occurs in basal conditions without an immune challenge^42^, while what we assessed in the brain was in response to a demyelination injury.

Blockade of GABA_A_R during late pregnancy augmented the density of microglial cells present in the vicinity of the demyelination lesion. This blockade had no significant effect on astrocytosis. Thus, GABA_A_-R blockade is cell specific as it appears to specifically target microglia. Despite this enhanced microglial cell density, we observed no significant change in inflammatory cytokines. Activated microglia perform two different functions; e.g. phagocytosis to clear myelin debris and secretion of inflammatory/regulatory molecules. This observation suggests that the GABA_A_R blockade affects negatively the intrinsic phagocytic action of microglia without significantly altering their inflammatory cytokines production. This potential dissociation between inflammatory and phagocytic functions of microglia is in line with previous study^43^.

GABA_A_R containing γ2 subunit shows more sensitivity to the action of ALLO^24^. In our hands, we did not detect the expression of γ2 subunit in the saline-injected corpus callosum of pregnant, virgin, or postpartum rats. Following demyelination however, γ2 immunopositive cells were mostly observed at the core of the lesion of the three experimental groups. Interestingly, the expression of γ2 subunit in the demyelinated corpus callosum of pregnant rats was significantly increased compared to virgin and postpartum animals. GABA_A_Rγ2 was observed in both OPCs and microglia, suggesting that these cell types can be targeted by ALLO-activated GABA_A_R. While both OPCs and microglia express GABA_A_Rγ2, it appears that GABAergic activation of oligodendrocytes is likely the primary mediator of enhanced remyelination during pregnancy. As alluded to earlier, the activation of GABA_A_R promotes the cellular proliferation of OPCs^39^. Therefore, it is possible that upregulation of the γ2 subunit by OPCs mediates the increased proliferation of these cells in pregnant animals through the action of ALLO.

It should be noted that one of the limitations of the present experimental study is the inability to test whether the increased GABAergic tone during pregnancy reduces the onset of new demyelinating lesions as seen in MS patients. There are preclinical evidence that suggest the potential involvement of GABAergic system in the pathology of demyelinating conditions such as MS^23^. Dysregulation of GABAergic signaling has been implicated in MS pathology^10,11^. In fact, both GABA and the activity of its synthesizing enzyme glutamate decarboxylase (GAD) are reduced in the blood serum of MS patients^11,44^. Together with our experimental data, these preclinical findings suggest that targeting GABAergic activation could be a promising therapeutic strategy for demyelinating conditions.

## Conclusion

In this study, we provide experimental evidence to support a potential mechanism underlying the enhanced remyelination during pregnancy. The cellular, molecular and ultrastructural basis of this pro-myelinating condition was extensively explored in a focal demyelination model in the corpus callosum of rats. These neuroprotective effects are, at least in part, driven by the GABAergic system. This study sheds light on a pivotal role of GABA_A_R in promoting remyelination during pregnancy and opens research avenues for the potential clinical use of GABA_A_R activators in the treatment of demyelinating diseases.

## Methods

### Animals

Male and female Sprague Dawely rats were obtained from the Animal Resources Centre, Faculty of Medicine, Kuwait University. Rats had access to pelleted chow and water *ad libitum* and were kept under a 12-h light, 12-h dark cycle (light 7 a.m. – 7 p.m.). All experiments were performed in accordance with the guidelines on the humane handling of experimental animals as established by the Kuwait University Health Sciences Center, Animal Research Ethics committee. All rats’ experimental procedures were approved by the Animal Research Ethics committee at the Faculty of Medicine/Kuwait University. The data supporting this paper are included in the main paper and in the associated Supplementary Data.

### Stereotaxic surgeries

Rats underwent stereotaxic surgery to inject either lysolecithin or pyrogen-free saline solution into the corpus callosum as previously described^45^. Rats were sacrificed at either 3 days or 7 days post-lysolecithin injection, two time points which correspond to the peak of demyelination and the start of remyelination in this model respectively^46,47^. Rats were deeply anesthetized with 1.5 mg/kg of urethane [(i.p.), Sigma Chemical Co., St. Louis, MO, USA], and transcardially perfused with ice-cold phosphate-buffered saline (PBS) (NaCl: 137 mmol/L, KCl: 2.7 mmol/L, Na_2_HPO_4_: 10 mmol/L, KH_2_PO_4_: 1.8 mmol/L). SD 5 summarizes the timeline of the experiments conducted in this study.

### Luxol fast blue staining

Following transcardial perfusion, the brains were collected and post-fixed in 10% neutral-buffered formalin solution (Sigma Chemical Co., St. Louis, MO, USA) for 48 hours. The two hemispheres of each brain were separated and embedded in paraffin (Sigma Chemical Co., St. Louis, MO, USA). The brains were cut sagittally at a 5 µm thickness and mounted on silane-coated slides (Superfrost Plus Micro Slide, VWR, Arlington Heights, IL, USA). Histologic monitoring of myelin was performed using Luxol fast blue staining as previously described^45^. Images of the lesion area were acquired with a camera-equipped light microscope (Axio Observer A1. Zeiss Microscope) using AxioVision software. Three brain sections from the center of the demyelination lesion of 4–7 different rats per group were acquired at 10x magnification and used for the image analysis. The demyelinated area was delineated and measured using ImageJ software^48^. The obtained values of lesion size were used as an index of de/remyelination as previously described^49^.

### Immunofluorescent staining

Brains were collected and fixed as described above. They were then embedded in paraffin, cut along the sagittal axis of the brain (5 µm) and processed for immuno-fluorescent detection of myelin, neurofilament, and markers of oligodendrocytes and oligodendrocytes precursor cells (OPC) as previously described^45,49^. Briefly, brain sections were incubated with primary antibodies overnight (see details in Table 1 in Supplementary File). On the following day, brain sections were washed 3 times with PBS solution and incubated with appropriate secondary antibodies tagged with either Alexa488 or Alexa555 (1:1000, Invitrogen, Carlsbad, CA, USA) for 2 hours at room temperature. Sections were washed and mounted for observation under a confocal microscope (Zeiss LSM 700 META microscope) using either 40x or 63x objectives. The analysis area of different immunofluorescence investigations are illustrated in SD 6 and SD 7. For analysis of NF^+^ and MBP^+^ fibers, six different fields from both edges of the lesion was analyzed from 3 different sections for each animal. The selected fields were used for the measurement of the fraction area covered by NF^+^ fibers, the fraction area covered by MBP + fibers, and the fraction area covered by juxtaposed NF^+^ and MBP^+^ fibers. Juxtaposition of NF^+^ and MBP^+^ fibers was determined using ImageJ software using the “RG2B_Colocalization” plug-in. The myelination index was calculated by dividing the value of juxtaposed NF^+^ and MBP^+^ fraction by the value of NF + fraction^50,51^.

### Transmission electron microscopy

In order to explore the ultrastructural changes in the myelin sheath following demyelination, transmission electron microscopy (TEM) was performed as previously described^45,52^. Semi-thin sections were stained with toluidine blue (SD 8) and examined under Zeiss Axio Observer A1 microscope to confirm the presence of the lesion area in the collected tissue. The ultrastructure of myelin was examined using a 10000x objective (JEOL’s JEM-1200 EXII Scanning Transmission Electron Microscope, Tokyo, Japan). The g-ratio of the remyelinated axons was calculated by dividing the diameter of the axon without the myelin sheath by the diameter of the axon with the myelin sheath^53^. A decrease in the g-ratio is indicative of a better remyelination process^36^. A total of 813 axons were analyzed in 88 fields from 3–4 animals in each animal group. Sixty to eighty axons per rat were used for these analyses.

### Western blot

Western blot was performed as previously described^45^. Briefly, rats were transcardially perfused with ice-cold PBS and the demyelinated area of the corpus callosum (~2 mm × 2 mm) was collected and snap-frozen in liquid nitrogen. The brain tissues were homogenized and the proteins levels were assayed using bicinchoninic acid protein assay, separated using a 12% SDS-PAGE gel, transferred to a nitrocellulose membrane, and incubated with the primary antibodies of interest overnight as previously described^52^ (for more details about the antibodies, see Table 1 in Supplementary File). The membranes were then washed and incubated with the appropriate horseradish-peroxidase tagged secondary antibody (Santa Cruz Biotechnology, Santa Cruz, CA, USA) for 2 hours at room temperature. The membranes were subsequently exposed to an enhanced chemiluminescence solution, and protein bands were detected on Kodak X-Omat film (SD 9, SD 10, SD 11 and SD 12). The optical density of each protein band was determined using ImageJ^48^ and expressed as a ratio of actin.

### Multiplex Enzyme-linked Immunosorbent Assay

The inflammatory cytokines in the lesioned corpus callosum were measured using a Luminex assay (RECYTMAG -60K; EMD Millipore Corporation, Massachusetts, USA). This assay allows the measurement of multiple cytokines simultaneously in a small volume of sample (i.e. the demyelinated corpus callosum). The lesioned area of the corpus callosum was visualized under the magnifier (2x), collected using a surgical blade and snap-frozen in liquid nitrogen. The collected tissue was then stored in −80 °C until use. At the time of the assay, brain tissues were homogenized and proteins were extracted and used in the assay. Each sample was assayed in duplicates and was tested for 2 pro-inflammatory (IL-1 β, TNFα) and 2 anti-inflammatory cytokines (IL-4 and IL-10). The detection sensitivities of the assays are 2.8 pg/mL for IL-1β, 1.9 pg/mL for TNF-α, 3.1 pg/mL for IL-4, and 2.7 pg/mL for IL-10. The inter-assay variability is 11.3% for IL-1β, 10.8% for TNF-α, 10.7% for IL-4, and 9.0% for IL-10. The intra-assay variability is 3.6% for IL-1β, 2.7% for TNF-α, 3.1% for IL-4, and 3.8% for IL-10. Cytokine concentrations were calculated using Luminex Manager Software (Luminex Software Inc., Riverside, CA, USA).

#### Experiment 1: Assessment of the extent of de/remyelination following experimental demyelination in virgin, pregnant, and postpartum female rats

Three months old female rats were housed 2 per cage. A proven male breeder was introduced during the dark cycle. The following day, a vaginal smear was taken and observed under a light microscope. The day of sperm detection in the vaginal smear was considered as gestational day 0 (GD0). Pregnant female rats were then housed individually. On GD13, randomly selected pregnant rats were given a stereotaxic injection of either lysolecithin or pyrogen-free saline solutions into the corpus callosum as described above.

A different group of pregnant rats were allowed to deliver their pups. Dams were then separated from their litters on the day of delivery to minimize the potential impact of post parturition-associated hormones on demyelination^32,54^. Focal demyelination was induced on the third day postpartum. Randomly selected postpartum rats were given a stereotaxic injection of either lysolecithin or a pyrogen-free saline solution into the corpus callosum. A separate group of virgin and sexually mature female rats were subjected to stereotaxic injection of either lysolecithin or pyrogen-free saline into their corpus callosum as described above. This part of the study comprises a total of six experimental groups as summarized in Table 2 (Supplementary File).

#### Experiment 2: Exploration of the effect of GABAAR blockade on remyelination in pregnant animals

Bicuculline (Bic) is a competitive antagonist of GABA_A_R^55^. This antagonist has been shown to specifically block GABA_A_R when given intraperitoneally at a dose of 1 mg/kg^56^. Therefore, pregnant animals with a demyelination injury in the corpus callosum received daily intraperitoneal injections of either Bic (1 mg/kg, dissolved in pyrogen-free saline) or an equivalent volume of pyrogen-free saline solution from the GD13 until GD20.

#### Experiment 3: Investigation of the effect of ALLO blockade during late pregnancy on remyelination in pregnant rats

Finasteride (Fin) is a selective blocker of 5α-reductase, a key enzyme in the production of ALLO^8,57^. A separate rat breeding was performed. On GD13, rats were subjected to focal demyelination as described earlier and given subcutaneous injections of Fin (100 mg/kg, dissolved in 85% sesame oil and 15% ethanol) starting at 2 hours after the demyelination insult. Subsequently, Fin was administered daily from the day of demyelination induction (GD13) until the day of sacrifice (GD20). The control group was subjected to focal demyelination and received daily equivalent volume subcutaneous injections of the vehicle solution (85% sesame oil and 15% ethanol) from GD13 until GD20.

### Statistical analysis

Data related to demyelination in peripartum and virgin female rats were compared using a one way-ANOVA followed by a Student-Newman Keuls *post-hoc* test. Data related to the effects of bicuculline and finasteride were compared using unpaired student’s *t* test. Data related to measurement of inflammatory cytokines were analyzed using a two-way ANOVA followed by a Student-Newman Keuls *post-hoc* test. Statistical significance is declared when the *p* value was less than 0.05.

## Supplementary information

Supplementary data
