# Supplementary material for: Enhanced remyelination during late pregnancy: involvement of the GABAergic system
Source: Sci Rep. 2019 May 22;9:7728. doi: 10.1038/s41598-019-44050-4 (PMC6531481; doi:10.1038/s41598-019-44050-4)
Supplement: Supplementary file 1 — Supplementary data [file 41598_2019_44050_MOESM1_ESM.pdf]

Enhanced remyelination during late pregnancy: involvement of the GABAergic system.

Samah Kalakh and Abdeslam Mouihate*
Department of Physiology, Faculty of Medicine, Kuwait University, P.O. Box 24923, Safat 13110, Kuwait

*Corresponding author
Abdeslam Mouihate
Department of Physiology
Faculty of Medicine
Health Sciences Centre
Kuwait University
e-mail: abdeslam@hsc.edu.kw
Phone: 965 2498 6363
Fax: 965 2533 8937

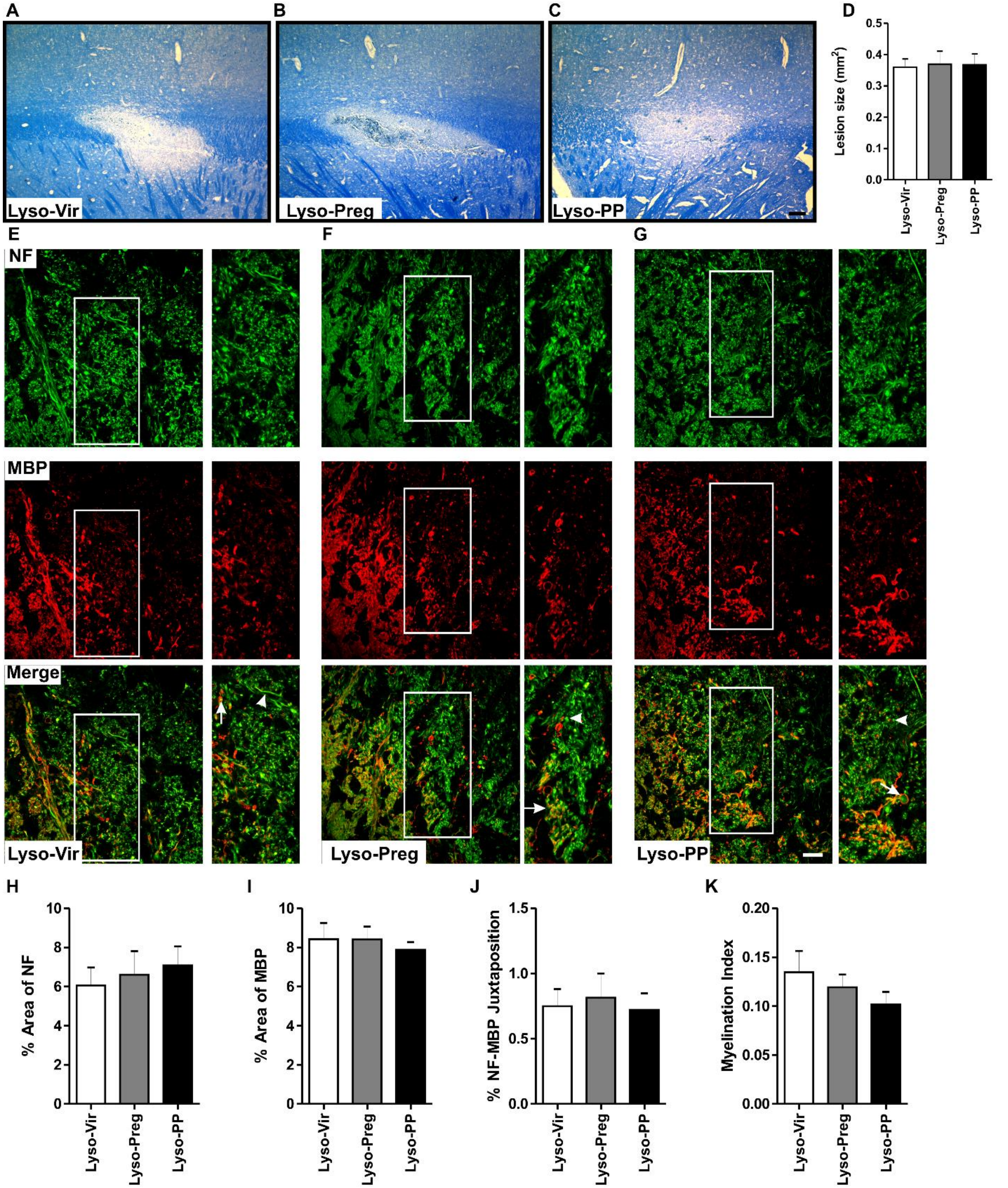

Supplementary data 1: The extent of demyelination lesion is not different between virgin, pregnant, and postpartum animals at the peak demyelination 3 days post-lysolecithin injection.

Images in (**A**), (**B**) and (**C**) show LFB staining of lysolecithin-injected corpora callosa of virgin, pregnant, and postpartum animals respectively. **D)** Bar graph shows that the demyelination lesion size was not significantly different between the three lysolecithin-

injected groups (Lyso-Vir: n=9, Lyso-Preg: n=8, Lyso-PP: n=8, $p>0.05$). (**E**), (**F**) and (**G**) show immunofluorescent images of NF (green) and MBP (red) at the edge of the demyelination lesion in virgin, pregnant, and postpartum animals respectively. Arrowheads indicate unmyelinated axons while arrows indicate myelinated ones. There was no significant difference in either the percentage area covered by NF^+ fibers **(H)**, the percentage area covered by MBP^+ fibers (**I**), the percentage of juxtaposed NF^+ and MBP^+ fibers (**J**), or the myelination index (**K**) between the three experimental groups ($p>0.05$). Data is presented as mean ± SEM. Scale bar in LFB staining = 200 µm. Scale bar in immunofluorescence images = 50 µm.

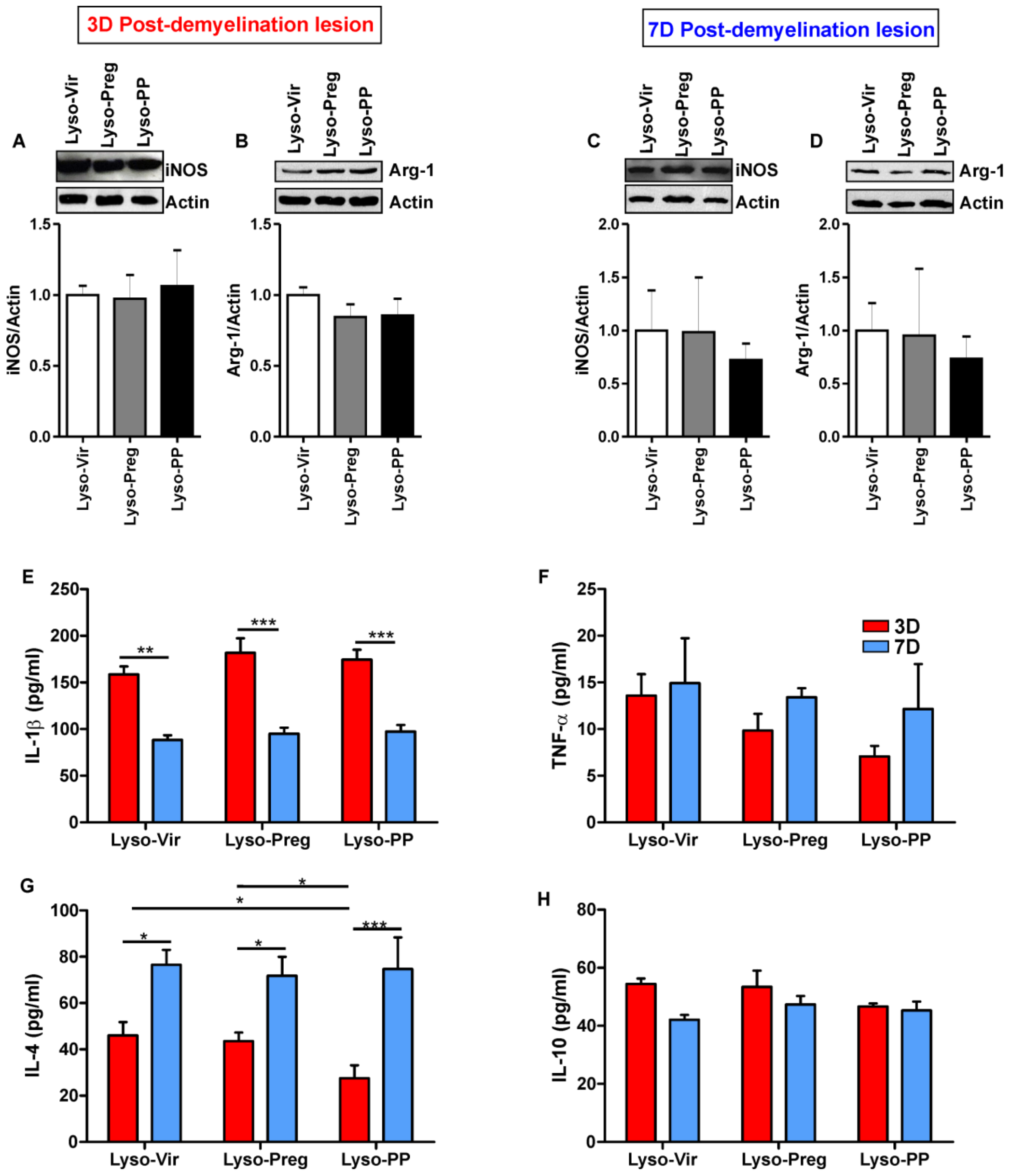

Supplementary data 2: Evaluation of inflammatory milieu within the demyelination lesion.

Microglial activation markers were investigated using western blot. The activation markers iNOS and Arg-1 were used to assess classically activated and alternatively activated micrgola respectively at 3D **(A, B)** and 7D **(C, D)** post-demyelination lesion. The expression levels of iNOS and Arg-1 were not different between pregnant, virgin, or post-partum rats neither at 3D nor at 7D post-demyelination lesion. **E-H** are bar graphs showing the cytokine measurements in the demyelinated corpus callosum tissue of virgin, pregnant, and postpartum rats 3 days (3D) and 7 days (7D) post-demyelination as assessed by multiplex ELISA [3D (Lyso-Vir: n=7, Lyso-Preg: n=8, Lyso-PP: n=8), 7D

(Lyso-Vir: n=5, Lyso-Preg: n=5, Lyso-PP: n=4)]. **E**) Levels of IL-1β are significantly downregulated 7 days post-demyelination relative to 3 days post-demyelination in virgin ($p<0.01$), pregnant ($p<0.001$), and postpartum ($p<0.001$) rats. **F**) TNF-α levels are not different between the three experimental groups at any time point ($p>0.05$). **G**) IL-4 significantly increased 7 days post-demyelination compared to 3 days post-demyelination in virgin ($p<0.05$), pregnant ($p<0.05$), and postpartum ($p<0.001$) rats. IL-4 levels were significantly lower in postpartum animals compared to virgin and pregnant rats ($p<0.05$). **H**) IL-10 levels are not different between the three experimental groups at any time point ($p>0.05$).

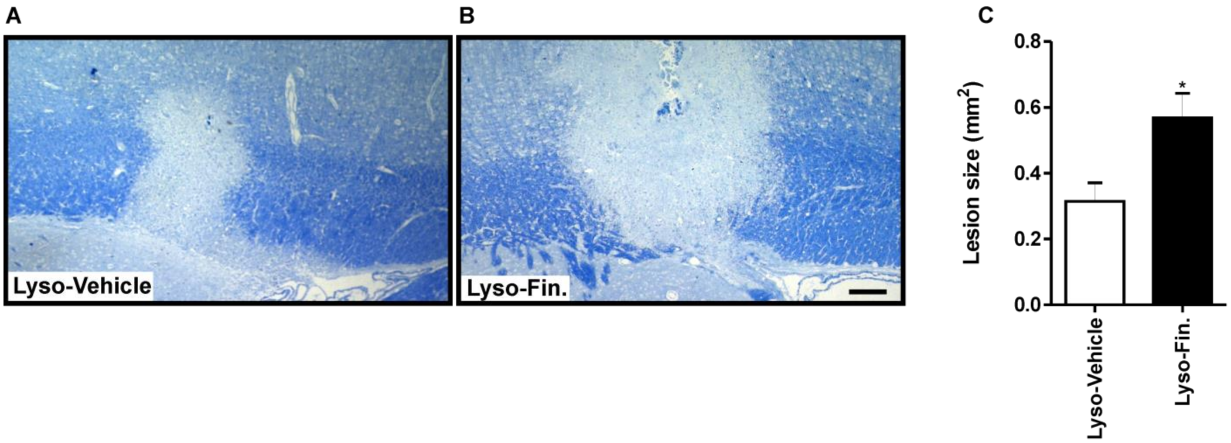

Supplementary data 3: Systemic Fin administration increases the demyelination lesion size in the corpus callosum of pregnant rats.

(A) and **(B)** show representative images of LFB staining in lysolecithin-injected corpus callosum of vehicle-treated and Fin-treated pregnant rats. **C)** Systemic administration of Fin resulted in a significantly larger demyelination lesion when compared to vehicle-treated rats (Lyso-Vehicle: n=5, Lyso-Fin: n=7, $p<0.05$). Results are represented as mean ± SEM. Scale bar = 200µm.

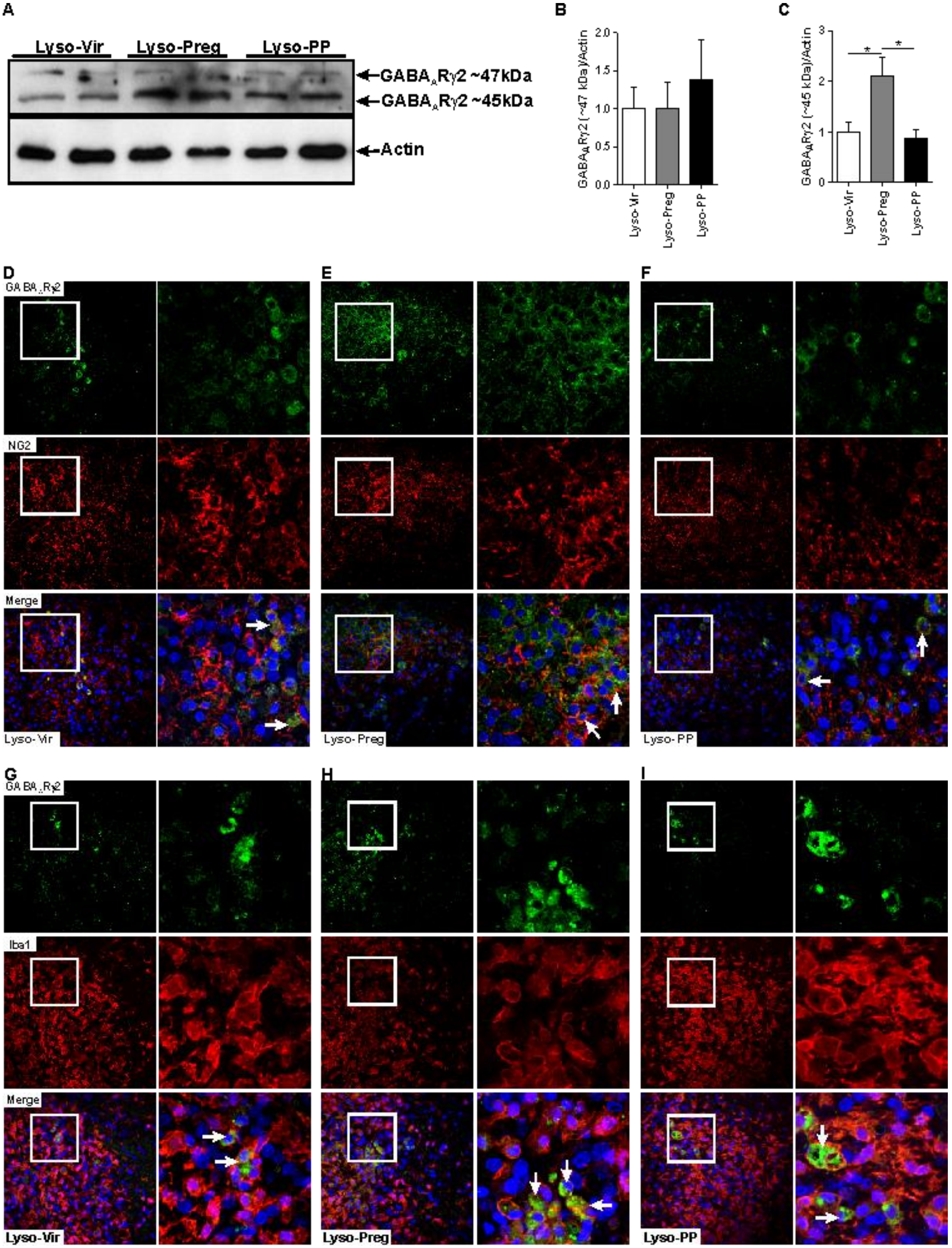

A
Lyso-Vir
Lyso-Preg
Lyso-PP
GABAARγ2 ~47kDa
GABAARγ2 ~45kDa
Actin
B
GABAARγ2 (~47 kDa)/Actin
C
GABAARγ2 (~45 kDa)/Actin
D
E
F
GABAARγ2
NG2
Merge
Lyso-Vir
Lyso-Preg
Lyso-PP
G
H
I
Iba1

Supplementary data 4: The expression of $GABA_AR\gamma2$ is upregulated in pregnant rats following demyelination in the corpus callosum.

A) Two isoforms of $GABA_AR\gamma2$ were detected at ~47 kDa and ~45 kDa. **B)** There was no significant difference in the expression level of the ~47 kDa isoform between the three experimental groups (Lyso-Vir: n=4, Lyso-Preg: n=4, Lyso-PP: n=4, $p>0.05$). **C)** Pregnant animals showed significantly higher expression of the ~45 kDa isoform compared to either virgin or postpartum animal groups ($p<0.05$). **(D-F)** $GABA_AR\gamma2$ (green) was immuno-detected in combination with the OPC marker NG2 (red). A fraction of $NG2^+$ cells co-expressed $GABA_AR\gamma2$ (arrows) in virgin **(D)**, pregnant **(E)**, and postpartum **(F)** animals. DAPI (blue) was used to visualize cell nuclei. **(G-I)** $GABA_AR\gamma2$ (green) was immuno-detected with the microglial marker Iba1 (red). A fraction of $Iba1^+$ cells co-expressed $GABA_AR\gamma2$ (arrows) in virgin **(G)**, pregnant **(H)**, and postpartum **(I)** animals. DAPI (blue) was used to visualize cell nuclei. Data is presented as mean ± SEM. Scale bar = 50µm.

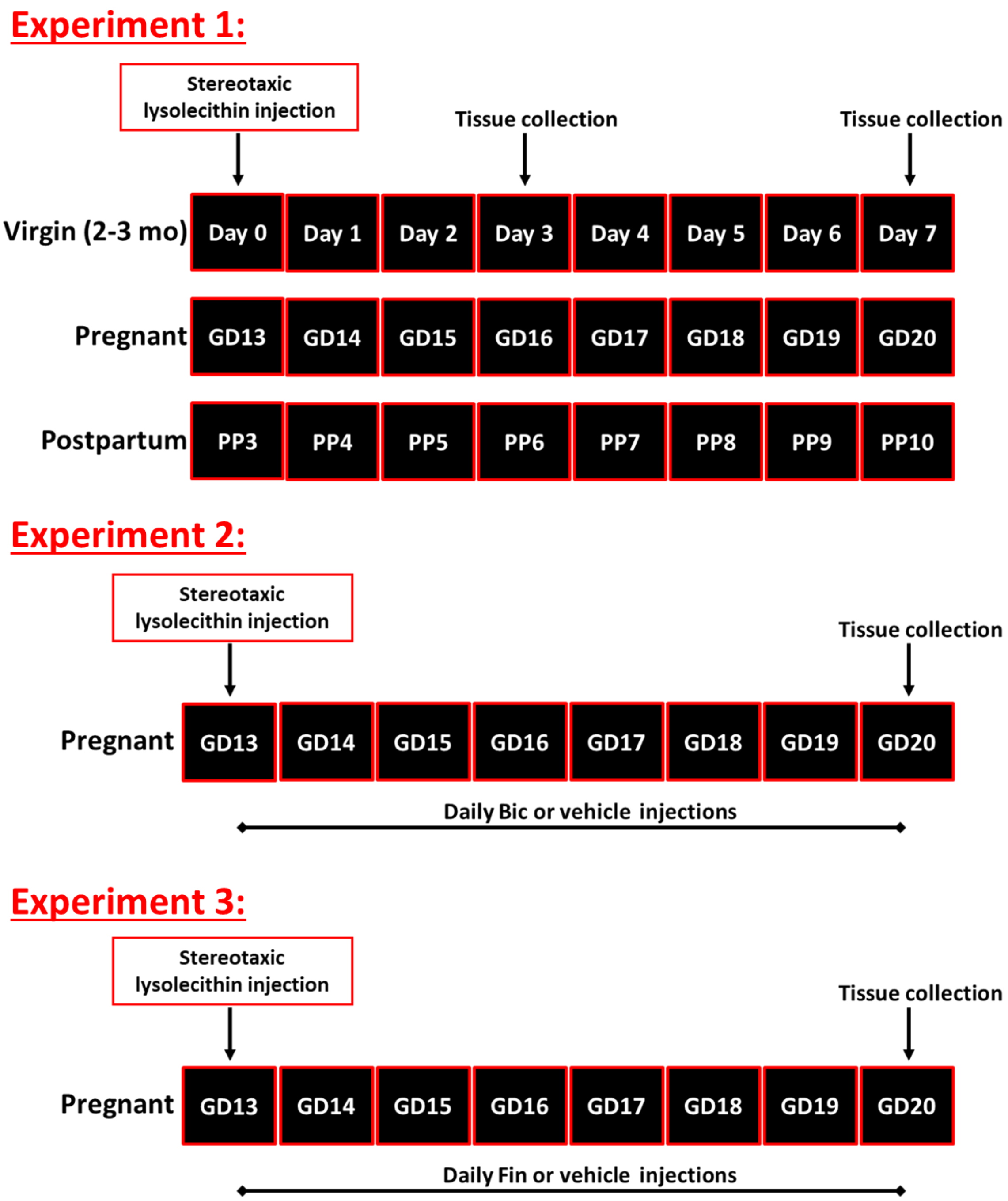

Supplementary data 5: Experimental design.
Stereotaxic surgery was performed to inject lysolecithin into the corpus callosum to induce a local demyelination lesion. In experiment 1, this surgery was performed on virgin 2-3 months-old rats, pregnant rats at gestational day 13 (GD13), and postpartum rats at postpartum day 3 (PP3). Brains of these animals were collected at both day 3 post-surgery and day 7 post-surgery. Experiments 2 and 3 were performed on pregnant rats. Stereotaxic surgery was performed at GD13. Treatment with bicuculline (Bic) or finasteride (Fin) was administered daily from the day of the surgery until the day of sacrifice 7 days post-surgery.

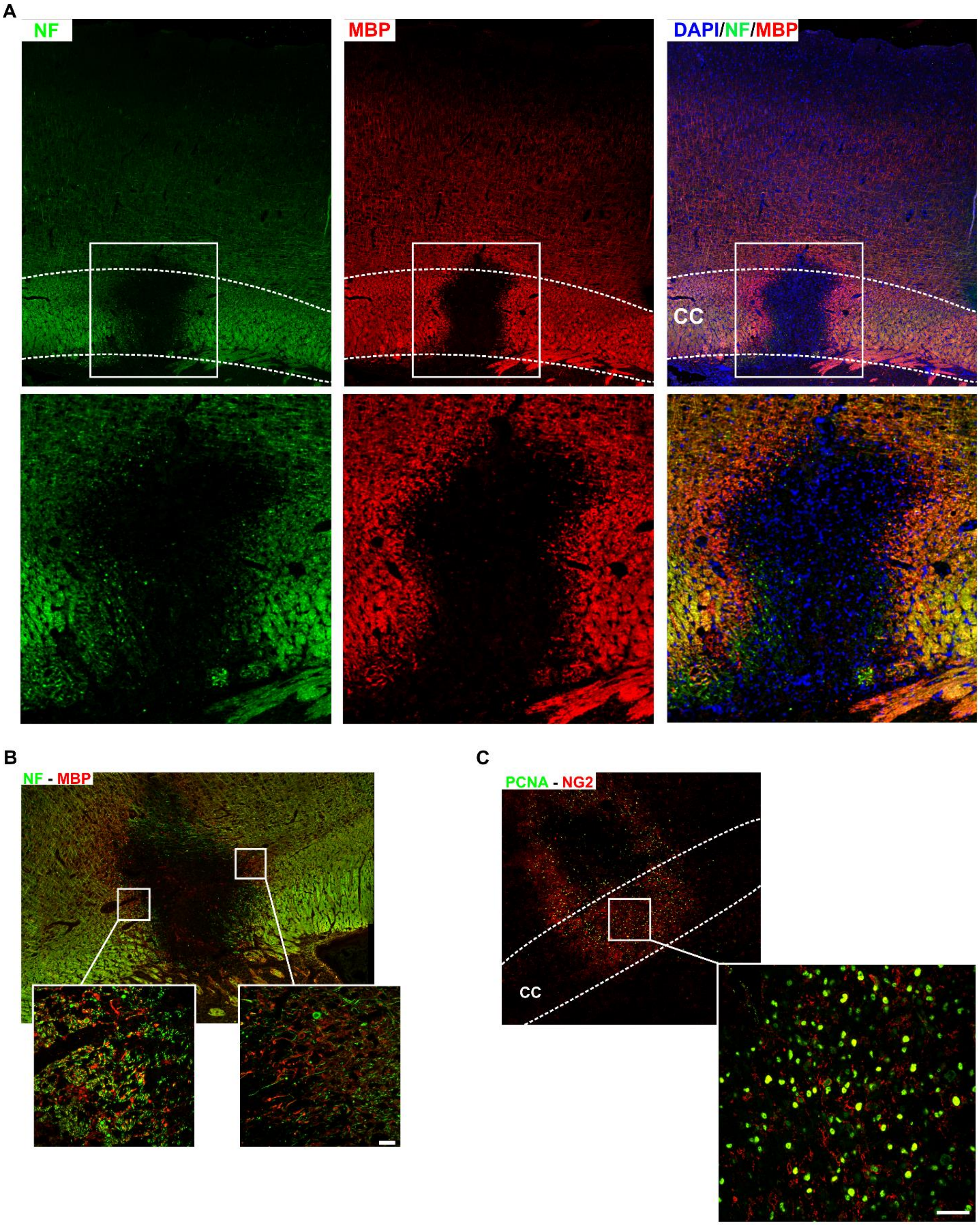
A
NF
MBP
DAPI/NF/MBP
CC
B
NF - MBP
C
PCNA - NG2
cc

Supplementary data 6: Lesion overview.

A) Immunofluorescent images showing an overview of the lysolecithin-injected corpus callosum (CC) 7 days post-lesion using DAPI, neurofilament (NF), and myelin basic protein (MBP). A series of images were aqcuired with 10x objective and stitched using the image J software (upper panel). The corpus callosum is outlined with the dashed lines. Lower panel shows a larger view of the demyelination lesion. The demyelination lesion is identified by the absence of MBP staining. Axonal loss at the centre of the lesion is evident as shown by the absence of NF staining. The centre of the lesion contains high density of DAPI. **B**) A series of immunoflourescent images of NF and MBP at 7 days post-lesion were acquired with 10x objective and stitched using the image J software. The two white boxes show an example of the sampled areas used for axonal integrity analysis at the edge of the lysolecithin-induced demyelination lesion. **C**) A series of immunoflourescent images of NG2 and PCNA at 7 days post-lesion were acquired with 10x objective and stitched using the image J software. The density of PCNA and NG2 is higher at the centre of the demyelination lesion. The white box shows an example of the area used for the evaluation of PCNA/NG2 density as well as PCNA/Olig2 density (see main manuscript). Scale bar = 50 µm.

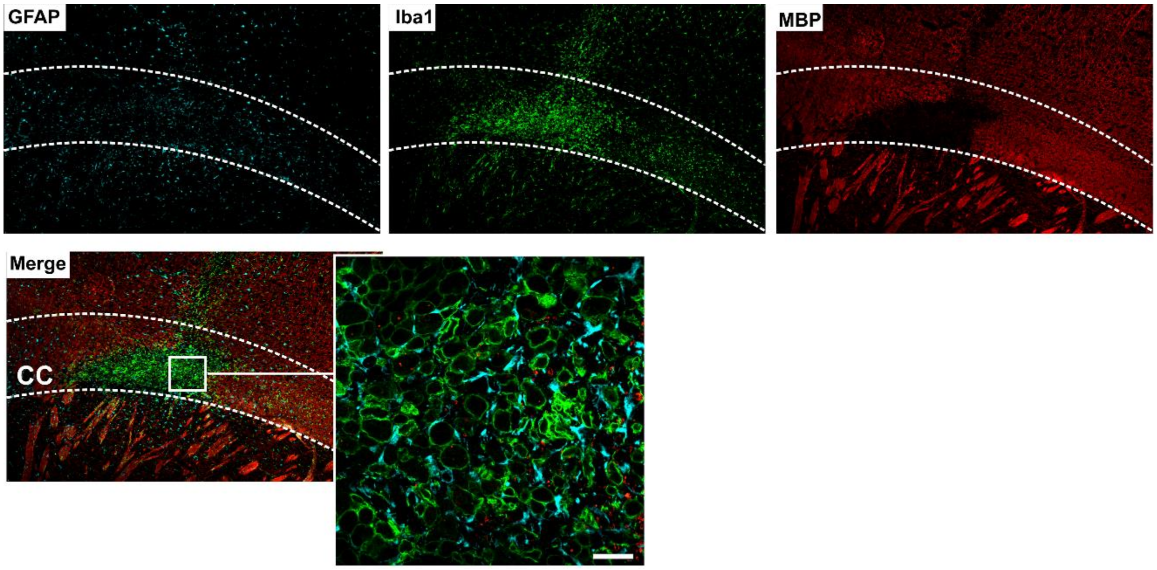

Supplementary data 7: Microglial activation and astrocytosis in the corpus callosum following lysolecithin-induced demyelination.
Immunofluorescent images showing the distribution of the astrocytic marker GFAP (blue), and the microglial marker Iba1 (green) 7 days post-lesion. The staining was combined with MBP (red) to identify the demyelinated area of the corpus callosum. Activated astrocytes were found both at the center and the edges of the demyelination lesion. Activated microglia were mainly located at the center of the lesion. The white box shows an example of the area used for analysis of astrocytic and microglial activation. Scale bar = 50 µm.

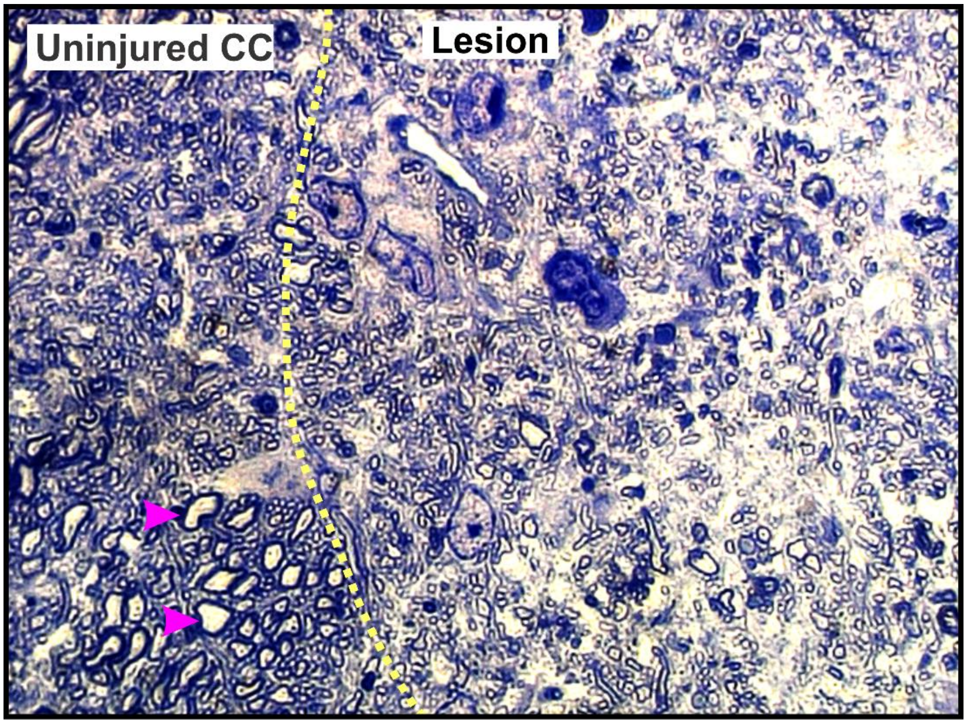

Supplementary data 8: Lesion confirmation using toluidine blue.
Representative image showing toluidine blue staining in semi-thin sections obtained to confirm the presence of the demyelination lesion in the tissue collected for TEM. The dashed line separates the lesioned and non-lesioned corpus callosum. The non-lesioned area has multiple myelinated axons consistent with the appearance of white matter tracts (arrowheads). The lesion area has reduced number of these myelinated axons.

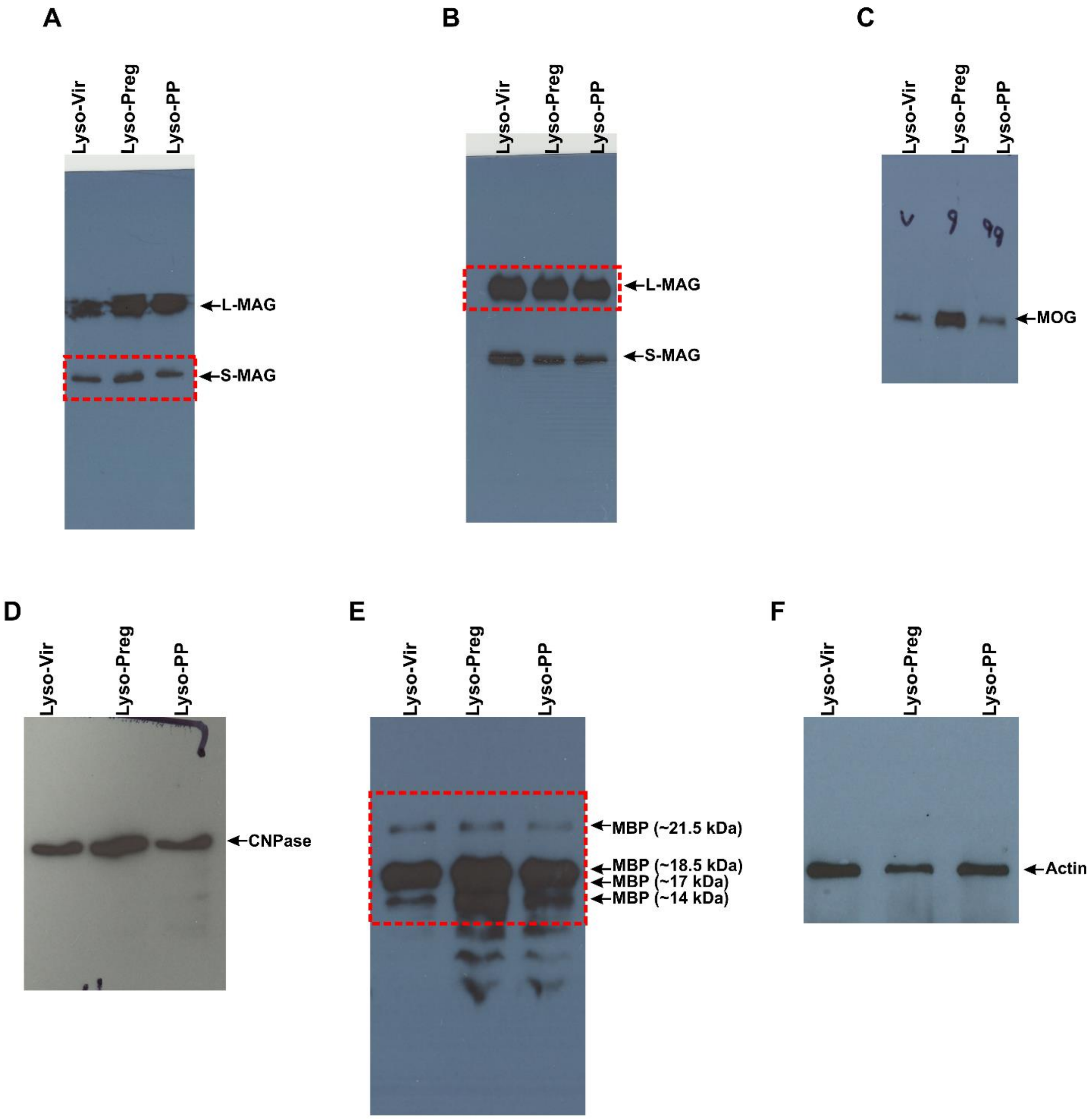

Supplementary data 9: Full-length blots for figure 3.

A) S-MAG full blot. Red dotted box shows the S-MAG band shown in figure 4. **B)** L-MAG full blot. Red dotted box shows the L-MAG band shown in figure 4 **C)** MOG full blot. **D)** CNPase full blot. **E)** MBP full blot. **F)** Actin full blot.

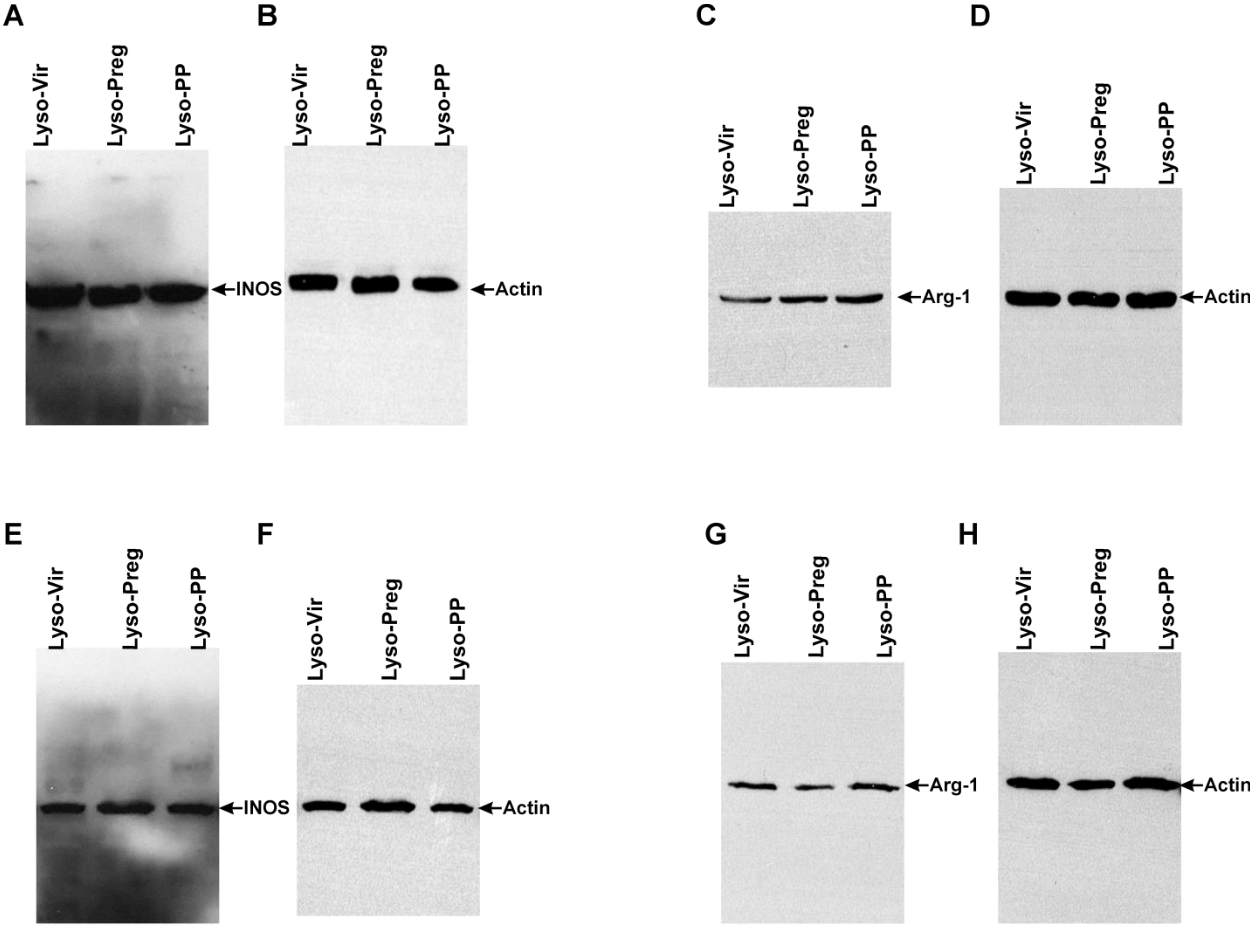

Supplementary data 10: Full-length blots for supplementary data 2.

(A) shows iNOS full blot assessed at 3D post-demyelination. **(B)** shows the full bot of actin used for iNOS normalization 3D post-demyelination. **(C)** shows Arg-1 full blot measured 3D post-demyelination. **(D)** shows the full bot of actin used for Arg-1 normalization 3D post-demyelination. **(E)** shows iNOS full blot measured 7D post-demyelination. **(F)** shows the full bot of actin used for iNOS normalization 7D post-demyelination. **(G)** shows Arg-1 full blot measured 7D post-demyelination. **(H)** shows the full bot of actin used for Arg-1 normalization 7D post-demyelination.

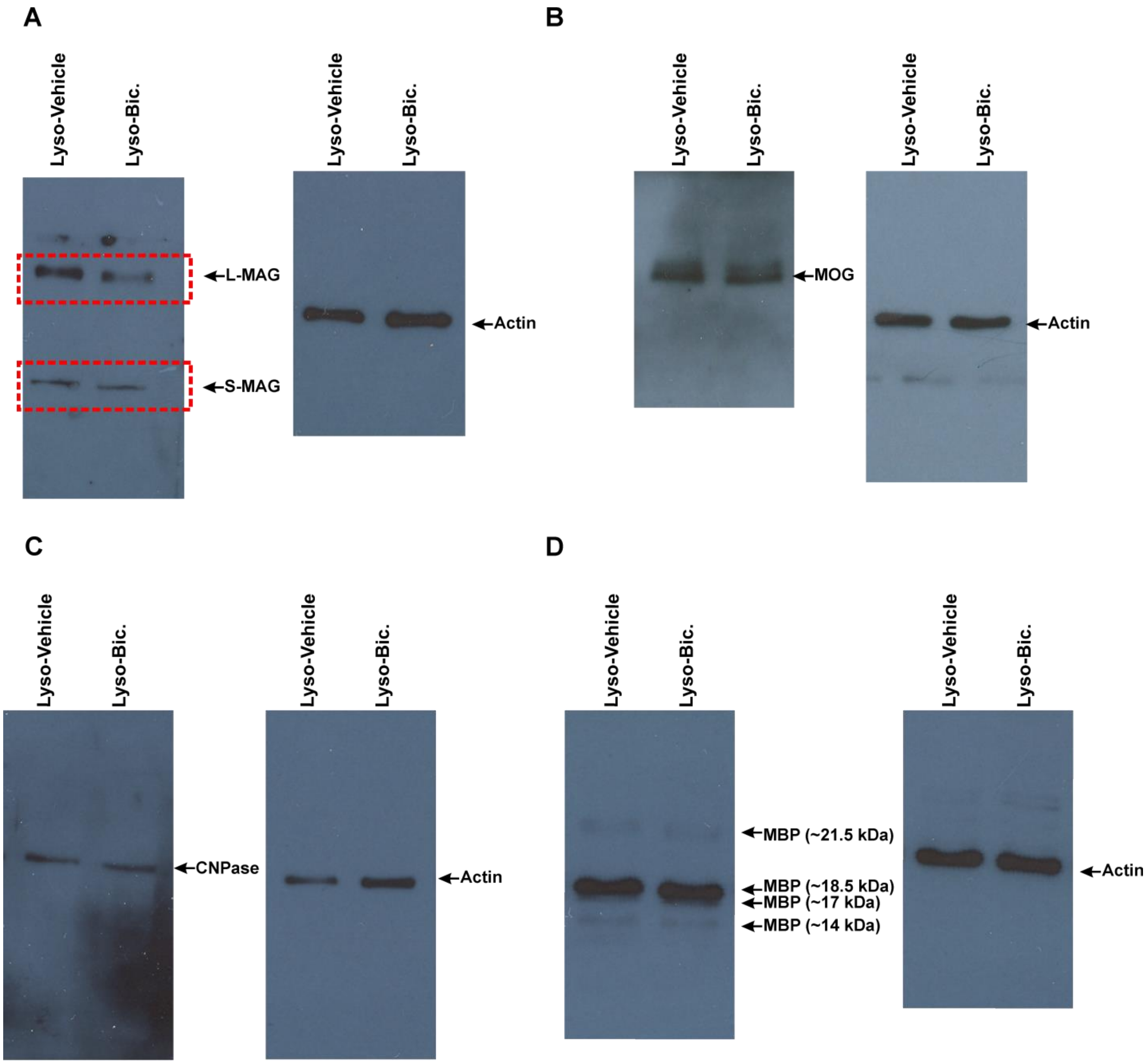

Supplementary data 11: Full-length blots for figure 7.

A) Left panel shows MAG full blot. Upper red dotted box shows the L-MAG band shown in figure 7. Lower red dotted box shows the S-MAG band shown in figure 7. Right panel shows the full bot of actin used for MAG normalization. **B)** Left panel shows MOG full blot. Right panel shows full blot actin used for MOG normalization. **C)** Left panel shows CNPase full blot. Right panel shows full blot of actin used for CNPase normalization. **D)** Left panel shows MBP full blot. Right panel shows full blot actin used for MBP normalization.

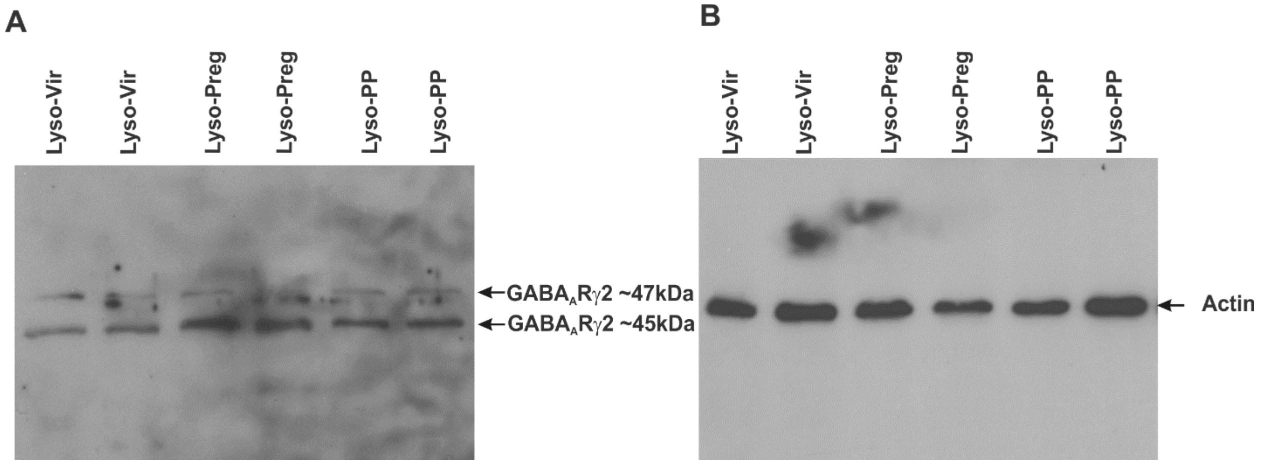

Supplementary data 12: Full-length blots for supplementary data 4.

(A) shows $GABA_A R\gamma 2$ full blot assessed at 7D post-demyelination. Two isoforms of $GABA_A R\gamma 2$ were detected at ~47 kDa (upper band) and ~45 kDa (lower band). **(B)** shows the full bot of actin used for $GABA_A R\gamma 2$ normalization 7D post-demyelination.

Table 1. Primary antibodies used in immunofluorescence and western blot.

Primary antibodies	Dilution	Company	Secondary antibodies
Rabbit polyclonal antibody anti-NG2	1:500	Millipore, MA, USA	Alexa Fluor 555 donkey anti-rabbit IgG
Mouse monoclonal antibody anti-PCNA	1:5000	Abcam, Cambridge, MA, USA	Alexa Fluor 488 donkey anti-mouse IgG
Rabbit monoclonal antibody anti-Olig2	1:200	Abcam, Cambridge, MA, USA	Alexa Fluor 488 donkey anti-rabbit IgG
Goat polyclonal antibody anti-neurofilament-M	1:1000	Santa Cruz Biotechnology, Santa Cruz, CA, USA	Alexa Fluor 488 donkey anti-goat IgG
Mouse monoclonal antibody anti-myelin basic protein	1: 2000	Calbiochem, Billerica, MA, USA	Alexa Fluor 555 donkey anti-mouse IgG
Goat polyclonal antibody anti-Iba1	1: 2000	Abcam, Cambridge, MA, USA	Alexa Fluor 488 donkey anti-goat IgG
Rabbit polyclonal antibody anti-GFAP	1: 2000	Sigma-Aldrich,St. Louis, Mo., USA	Alexa Fluor 405 donkey anti-rabbit IgG
Mouse monoclonal antibody anti-Anti-$GABA_A$ γ2 Subunit	1:400	Millipore, MA, USA	Alexa Fluor 488 donkey anti-mouse IgG
Mouse monoclonal antibody anti-MAG	1:1000	Abcam, Cambridge, MA, USA	HRP-conjugated Donkey anti-mouse IgG
Mouse monoclonal antibody anti-MOG	1:1000	Millipore, MA, USA	HRP-conjugated Donkey anti-mouse IgG
Mouse monoclonal antibody anti-CNPase	1:2000	Sigma-Aldrich,St. Louis, Mo., USA	HRP-conjugated Donkey anti-mouse IgG
Mouse monoclonal antibody anti-myelin basic protein	1: 5000	Calbiochem, Billerica, MA, USA	HRP-conjugated Donkey anti-mouse IgG
Mouse monoclonal antibody anti-Anti-$GABA_A$ γ2 Subunit	1:1000	Millipore, MA, USA	HRP-conjugated Donkey anti-mouse IgG
Rabbit polyclonal antibody anti-Actin	1: 5000	Sigma-Aldrich,St. Louis, Mo., USA	HRP-conjugated Donkey anti rabbit IgG
Mouse monoclonal antibody anti-iNOS	1:1000	BD Biosciences, San Jose, CA, USA	HRP-conjugated Donkey anti-mouse IgG
Mouse monoclonal antibody anti-Arg-1	1:1000	BD Biosciences, San Jose, CA, USA	HRP-conjugated Donkey anti-mouse IgG

Table 2. Experimental groups.

Experimental group	Abbreviation
Saline-virgin	Sal-Vir
Saline-pregnant	Sal-Preg
Saline-postpartum	Sal-PP
Lysolecithin-virgin	Lyso-Vir
Lysolecithin-pregnant	Lyso-Preg
Lysolecithin-postpartum	Lyso-PP
